# LC3 and STRAP regulate actin filament assembly by JMY during autophagosome formation

**DOI:** 10.1083/jcb.201802157

**Published:** 2019-01-07

**Authors:** Xiaohua Hu, R. Dyche Mullins

**Affiliations:** 1 Department of Cellular and Molecular Pharmacology, University of California, San Francisco, School of Medicine, San Francisco, CA; 2 Howard Hughes Medical Institute, Chevy Chase, MD

## Abstract

During autophagy, actin filament networks move and remodel cellular membranes to form autophagosomes that enclose and metabolize cytoplasmic contents. Two actin regulators, WHAMM and JMY, participate in autophagosome formation, but the signals linking autophagy to actin assembly are poorly understood. We show that, in nonstarved cells, cytoplasmic JMY colocalizes with STRAP, a regulator of JMY’s nuclear functions, on nonmotile vesicles with no associated actin networks. Upon starvation, JMY shifts to motile, LC3-containing membranes that move on actin comet tails. LC3 enhances JMY’s de novo actin nucleation activity via a cryptic actin-binding sequence near JMY’s N terminus, and STRAP inhibits JMY’s ability to nucleate actin and activate the Arp2/3 complex. Cytoplasmic STRAP negatively regulates autophagy. Finally, we use purified proteins to reconstitute LC3- and JMY-dependent actin network formation on membranes and inhibition of network formation by STRAP. We conclude that LC3 and STRAP regulate JMY’s actin assembly activities in trans during autophagy.

## Abstract (precis)

The actin regulator JMY creates filament networks that move membranes during autophagy. Hu and Mullins find that JMY is normally inhibited by interaction with the STRAP protein, but upon starvation, JMY is recruited away from STRAP and activated by LC3.

## Introduction

Autophagy is a catabolic process during which membranes move and remodel to form autophagosomes, organelles bounded by double membranes that engulf and metabolize cytoplasmic contents (Shibutani and Yoshimori, 2014). In 2015, Mi et al. (2015) found evidence that branched actin networks created by the Arp2/3 complex drive some of the membrane movements required for autophagosome formation. After this initial discovery, two groups identified a pair of related nucleation-promoting factors, WASP homologue associated with membranes and microtubules (WHAMM) and junction mediating and regulatory protein (JMY), as contributors to autophagosome biogenesis. Kast et al. (2015) found that WHAMM promotes assembly of actin networks on early autophagosomal membranes and that decreasing WHAMM expression reduces the number and size of autophagosomes. Coutts and La Thangue (2015) found that JMY promotes autophagy by directing actin assembly on membranes that also contain the autophagy regulator, LC3. They also noticed that the N-terminal region of JMY contains a consensus LC3-interacting region (LIR) found in many autophagy-related proteins. Intriguingly, removal of the N-terminal LIR not only disrupted the association of JMY with LC3-containing membranes, but also impaired JMY’s ability to create actin structures in the cytoplasm. The effect of N-terminal deletions on actin assembly is surprising, given that JMY’s previously described actin- and Arp2/3-binding sites are all located near the C terminus. Little is known about how upstream factors regulate WHAMM- or JMY-directed actin assembly, but the results of Coutts and Lathangue (2015) suggest that JMY’s N-terminal region might regulate both localization and nucleation activity.

JMY is an enigmatic actin regulator. Initially described as a coactivator of p53-mediated apoptosis after DNA damage (Shikama et al., 1999), as we later found, JMY contains an Arp2/3-activating sequence, called a WCA domain, common to class II nucleation promoting factors (Welch and Mullins, 2002; Zuchero et al., 2009). This WCA domain resides in the C-terminal region of JMY and comprises three conserved motifs: (1) a set of three actin-binding Wasp-homology 2 (WH2 or W) domains; (2) a central connecting domain (C) that binds both actin and the Arp2/3 complex; and (3) an acidic region (A) that binds the Arp2/3 complex. In addition to promoting Arp2/3-dependent nucleation, JMY’s three WH2 domains also nucleate actin filaments on their own (Zuchero et al., 2009) using a mechanism similar to that of spire (Quinlan et al., 2005). With the possible exception of Las17p in budding yeast (Urbanek et al., 2013), this combination of intrinsic and Arp2/3-mediated nucleation activities is unique to JMY, suggesting that its function might be highly specialized.

Although it can function as a coactivator of p53-dependent transcription in the nucleus, JMY localizes predominantly to the cytoplasm in unperturbed cells (Firat-Karalar et al., 2011; Schlüter et al., 2014), where it has been reported to participate in a variety of cellular processes in addition to autophagy, including cell migration and adhesion (Coutts et al., 2009), regulation of neurite outgrowth (Firat-Karalar et al., 2011), and asymmetric cell division of mouse oocytes (Sun et al., 2011). JMY has also been reported to interact with ER-resident protein VAP-A (Schlüter et al., 2014), which is important for autophagosome biogenesis, since the ER is a major source of membranes for autophagosome formation (Axe et al., 2008; Shibutani and Yoshimori, 2014; Zhao et al., 2018).

The regulation of JMY’s actin nucleation activity in the cytoplasm is less understood than the regulation of its nuclear functions. No modulators of JMY’s actin nucleation activity have been identified, whereas the acetyltransferase p300 and its binding partner stress-responsive activator of p300 (STRAP, also named TTC5) are known to interact with JMY in the nucleus. STRAP appears to act as a scaffold, facilitating formation of the JMY/p300 complex and preventing JMY’s degradation by MDM2 (Demonacos et al., 2001). Little is known about the cytoplasmic role of STRAP, including whether it interacts with JMY in the cytoplasm.

In the present study, we identify both positive and negative regulators of JMY’s actin nucleation activity in the cytoplasm. We show that LC3 directly recruits JMY to membrane surfaces and enhances its de novo actin nucleation activity via a cryptic actin-binding sequence near JMY’s N terminus. Because the nucleation activity of the Arp2/3 complex requires preexisting, “mother” filaments, the enhancement of JMY’s N-terminal, cryptic nucleation activity has a knock-on effect that dramatically enhances actin network formation. We also show that STRAP potently inhibits actin nucleation stimulated by JMY and antagonizes JMY activation by membrane-associated LC3. STRAP also turns out to be a previously unrecognized negative regulator of starvation-induced autophagy. In fed cells, JMY primarily associates with nonmotile, STRAP-containing vesicles. Upon starvation-induced autophagy, JMY localization shifts to LC3-containing membranes that move on polarized actin networks.

## Results

### JMY translocates to motile LC3-positive vesicles from nonmotile STRAP vesicles upon starvation-induced autophagy

**Figure 1.**
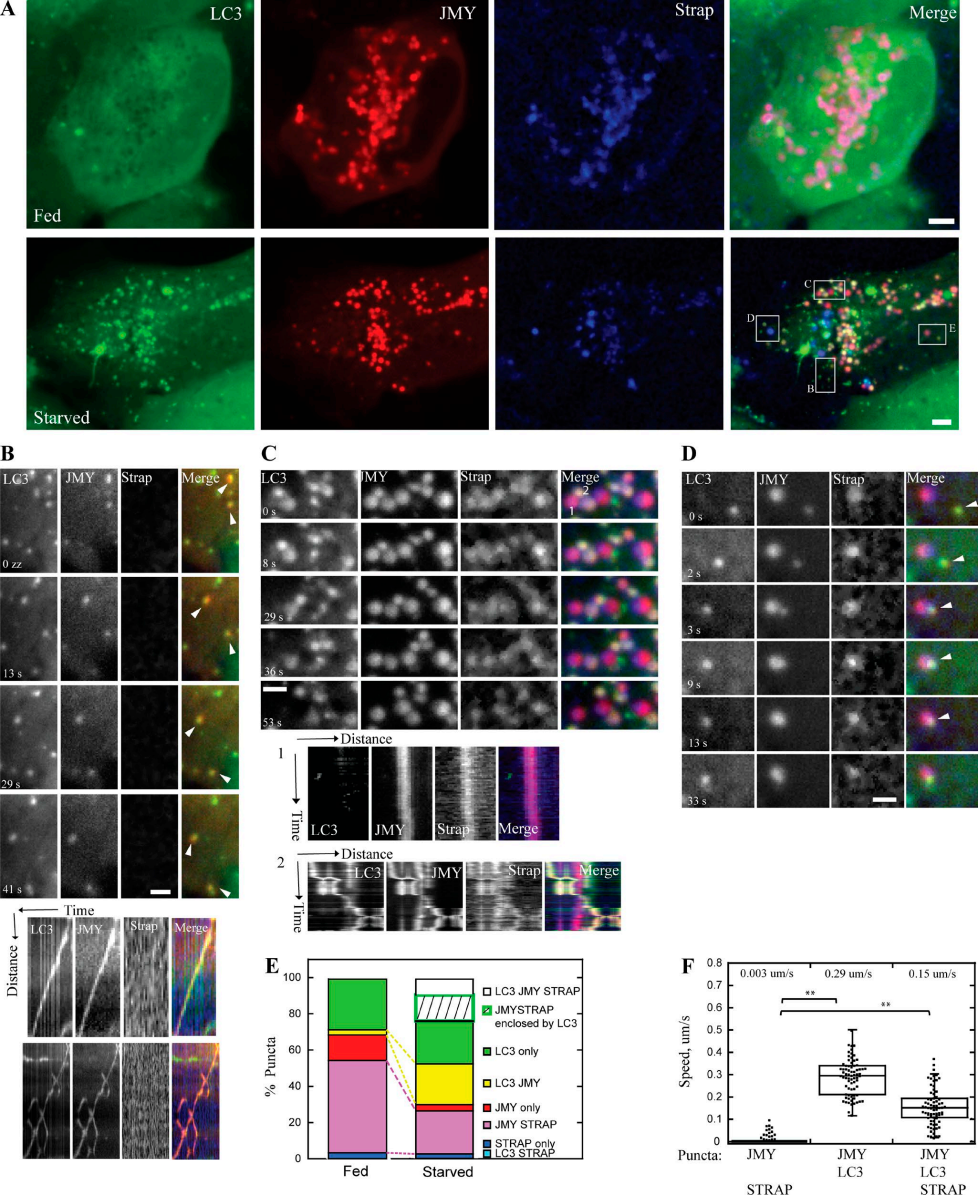
**In U2OS cells, JMY translocates from STRAP-positive nonmotile foci to motile LC3-positive motile foci upon starvation. (A)** JMY (red) and STRAP (blue) colocalize on foci under normally fed conditions (top). More JMY (red) colocalizes with LC3 (green) when cells are starved in HBSS (bottom). **(B)** Two vesicles (arrowheads) that contain both JMY and LC3 but no STRAP (yellow color in merge channel) move persistently through cytoplasm. Kymographs show travel distance versus time. **(C)** Enlarged box indicates two populations of vesicles. (1) Vesicles that contain JMY and STRAP but no LC3 (magenta color in merge channel) are nonmotile. Kymograph 1 shows one example of a nonmotile JMY- and STRAP-positive vesicle. (2) Vesicles that contain all three proteins, JMY, STRAP, and LC3 (yellow-white color in merge channel), move in a saltatory manner. Kymograph 2 shows one example of the saltatory movement of a JMY-, STRAP-, and LC3-positive vesicle. **(D)** JMY- and LC3-positive vesicle (yellow color in merge channel, arrowhead) moves and fuses with JMY- and STRAP-positive (magenta color in merge channel) vesicle. The fused vesicle is nonmotile during observation. **(E)** Quantification of colocalization of JMY, STRAP, and LC3 under fed and starved conditions. The percentage is normalized to total number of puncta (including all LC3, JMY, and STRAP puncta) of the corresponding cell (*n* = 3 independent experiments, 24 cells, total number of puncta per cell: 15 ~ 83). Quantifications of colocalization normalized to each protein are shown in Fig. S1 (A–C). **(F)** Quantification of migration speed of JMY-positive vesicles (number of JMY- and LC3-positive puncta: 73; number of JMY- and STRAP-positive puncta: 208; number of JMY-, LC3-, and STRAP-positive puncta: 73, *n* = 3, **, P < 0.01, Student’s *t* test. For all box plots: boxes represent upper and lower quartile with median value displayed as a line; the lines extending from each box mark the minimum and maximum of data set within acceptable range; the range is defined as quartile limit ±1.5 (*, interquartile distance). Temperature: 37°C. Scale bars: whole cell, 5 µm; zoom-in box, 2 µm.

To better understand the regulation of JMY’s activity in the cytoplasm, we coexpressed a JMY-mCherry fusion protein together with two JMY interacting proteins, GFP-LC3B and SNAP-tagged STRAP, in U2OS cells (Video 1). In normally fed cells, both JMY and STRAP localized primarily to cytoplasmic foci (Fig. 1, A and E; and Fig. S1, A–C), and many JMY foci also contained STRAP (81 ± 18%; Fig. S1 A). We observed that only a small fraction of LC3 vesicles also contained JMY (6.9 ± 13%; Fig. S1 B). Upon induction of autophagy by starvation, more JMY puncta accumulated LC3 (from 10 ± 20 to 30 ± 24%; Fig. S1 A), whereas the overlap between JMY and STRAP sharply decreased (from 81 ± 18% to 34 ± 21%; Fig. S1 A), consistent with a shift in JMY’s location and function upon starvation (Fig. 1, A and E; and Video 1). Moreover, vesicles containing JMY and LC3, but lacking STRAP, began moving rapidly and persistently through the cytoplasm (0.29 ± 0.08 µm/s; Fig. 1, B and F). In contrast, vesicles containing JMY and STRAP but lacking LC3 were nonmotile (0.003 ± 0.012 µm/s; Fig. 1, C [kymograph 1] and F).

LC3 and STRAP almost never colocalized in the absence of JMY (from 0% in fed cells to 0.8 ± 2% in starved cells; Fig. S1 B). Occasionally in starved cells, all three proteins, JMY, STRAP, and LC3, were present on the same puncta (12 ± 9%; Fig. S1 A), and these puncta exhibited saltatory movement (0.15 ± 0.08 µm/s; Fig. 1, C [kymograph 2] and F), suggesting that LC3 and STRAP were competing to bind JMY and antagonistically modulating its activity. We also observed that some JMY and STRAP puncta (19 ± 17%; Fig. S1 A) were enclosed in LC3 vesicles (Fig. S1, D and E), and that these were much less motile than other JMY-LC3 vesicles (Fig. S1 F). In rare cases, we observed JMY- and LC3-positive vesicles migrate toward and merge with nonmotile JMY- and STRAP-positive vesicles (Fig. 1 D), which might reflect a process in which LC3-containing membranes recruit JMY away from its interaction with STRAP.

**Figure 2.**
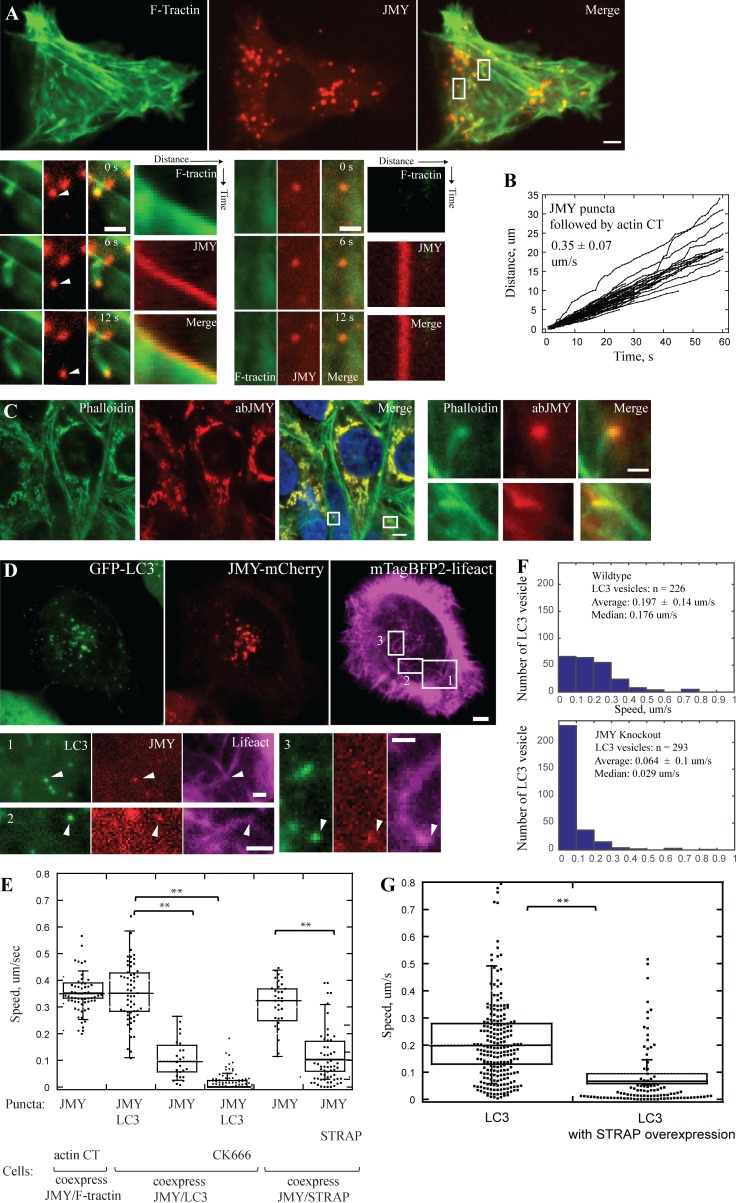
**In U2OS cells, LC3 and JMY vesicles move on polarized actin network. (A)** A subset of punctate JMY structures (red) are propelled through the cytoplasm (arrowheads) by actin comet tails (green) in fed U2OS cells. Enlarged boxes show one motile JMY focus with an associated actin comet tail (left) and one nonmotile JMY focus with no actin network (right). **(B)** Raw distance versus time plots of JMY-positive foci propelled by actin comet tails (63 puncta, 9 cells, *n* = 3 independent experiments). **(C)** Polarized actin networks (labeled by phalloidin, green) attach to endogenous JMY (JMY antibody, red) puncta in immunofluorescent staining in fed cells. **(D)** JMY- and LC3-positive vesicles are propelled by polarized actin networks in cells starved in HBSS. Enlarged boxes show actin networks (magenta) pushing JMY (red) and LC3 (green) positive vesicles (arrowhead) toward plasma membrane (1), toward nucleus (2), or in a more complex trajectory (“V” shaped movement; 3). **(E)** Quantification of migration speed of JMY-positive vesicles in cells expressing various sets of two proteins: JMY and F-tractin, JMY and LC3 (with or without CK666), JMY and STRAP (8∼11 cells, **, P < 0.01, Student’s *t* test). **(F)** Histograms show decrease in migration speed of LC3-positive vesicles in JMY knockout cell line (bottom) compared with WT U2OS cells (top). **(G)** The migration speed of LC3 vesicles decreases when U2OS cells overexpress STRAP (20 ~ 25 cells, *n* = 3 independent experiments, **, P < 0.01, Student’s *t* test). Temperature: 37°C. Scale bars: whole cell, 5 µm; zoom-in box, 2 µm.

We further confirmed the above result by coexpressing only two proteins at a time in U2OS cells: either GFP-LC3B and JMY-mCherry or JMY-mGFP and STRAP-mCherry. As in the experiments described above, JMY puncta displayed directed motility when colocalized with LC3 but little or no motility when colocalized with STRAP. Occasionally we also observed JMY and LC3 colocalized on membrane tubules (Figs. 2 E and S2 and Videos 2 and 3). Taken together, our data indicate that starvation triggers JMY to shift from nonmotile STRAP-containing membranes to motile, LC3-positive autophagosomes.

### JMY promotes formation of actin networks on motile, LC3-positive vesicles

Coutts and La Thangue (2015) found actin to be associated with LC3- and JMY-positive vesicles; thus we hypothesized that JMY might assemble actin comet tails to drive vesicle motility. To test this possibility, we coexpressed JMY-mCherry with a live-cell probe for filamentous actin (GFP-F-tractin) in U2OS cells. More than half of JMY puncta (66 ± 16%, 293 puncta, from nine cells) were associated with polarized actin comet tails, whereas the rest had no clear association with filamentous actin (Fig. 2 A). The actin-associated JMY foci moved rapidly through the cytoplasm, at an average speed of 0.35 ± 0.07 µm/s (Fig. 2, A, B, and E), apparently propelled by actin filament assembly (Video 4) in a manner similar to the motility of some intracellular pathogens such as *Listeria monocytogenes*. To test whether these motile puncta were created by overexpression of labeled JMY, we performed fluorescence microscopy on fixed U2OS cells stained with phalloidin and an anti-JMY antibody to visualize the distribution of endogenous JMY and actin. As in the live cell experiments, we observed polarized actin networks associated with endogenous JMY foci (Fig. 2 C), indicating that JMY-associated actin comet tails are not an overexpression artifact.

To verify that movement of LC3- and JMY-postive foci is driven by polarized actin comet tails, we coexpressed fluorescent derivatives of JMY and LC3 together with a fluorescent probe for filamentous actin (mTagBFP-lifeact) in U2OS cells. Using three-color fluorescence microscopy, we found that all motile, JMY- and LC3-positive vesicles were associated with actin comet tails (Fig. 2 D and Video 5). Slightly more than half of the motile LC3/JMY foci (56%) migrated toward the plasma membrane, whereas 21.9% migrated toward the nucleus, and the remainder moved in a more circumferential path around the cell. It is likely that these autophagosomes are moving along the ER tubule, because we observed JMY comigrate with its binding protein VAPA, an ER-resident protein involved in vesicle budding and membrane transport (Schlüter et al., 2014). In these experiments, the polarized actin network propelled JMY along the VAP-A–labeled ER tubule (Fig. S3). When we treated cells with CK666, which inhibits nucleation activity of Arp2/3 complex, all of the JMY- and LC3-positive puncta became nonmotile, with an average speed of 0.015 ± 0.028 µm/s (Fig. 2 E and Video 6), indicating that movement of JMY- and LC3-positive structures requires Arp2/3-mediated actin filament assembly.

To investigate JMYs role in promoting actin-driven movement of LC3-containing vesicles, we used CRISPR to edit the JMY gene locus and knockout JMY protein expression in our U2OS cells (Fig. S4, A and B). We observed that, upon starvation, the majority of LC3-positive puncta (75.2 ± 21%) in these JMY knockout cells remained nonmotile (Fig. 2 F and Video 7). We interpret this result as evidence that JMY-induced actin assembly drives motility of LC3-containing membranes.

We next treated U2OS cells with Bafilomycin A, to prevent acidification of mature autophagosomes and block autophagic flux (Yamamoto et al., 1998; Klionsky et al., 2008). Under these conditions, JMY/LC3-positive vesicles accumulated in the perinuclear region (Fig. S4 D). Interestingly, when we added the Arp2/3 inhibitor CK666 together with Bafilomycin A, JMY- and LC3-positive vesicles lost their perinuclear localization (Fig. S4, E and F), suggesting that, in addition to microtubule motors (Jahreiss et al., 2008; Pankiv and Johansen, 2010; Pankiv et al., 2010), actin-based movement may also be important for bulk centripetal flow of autophagosomes and/or detachment from their original sites of biogenesis.

### Colocalization of JMY with other autophagosomal markers

To better define JMY’s role in autophagy, we looked for colocalization with other membrane markers in normal and perturbed cells. An early step in autophagy is the phosphorylation of phosphatidyl inositol (PI) to form PI(3)P, which then recruits LC3. We found that blocking the activity of PI-kinases with wortmannin A disrupted the punctate localization of both LC3 and JMY (Fig. S5 A), suggesting that JMY recruitment is not upstream of PI(3)P accumulation. We did, however, observe colocalization and comigration of JMY with DFCP1 and ATG9, two proteins involved in the initiation and nucleation phases of phagophore assembly (Papinski et al., 2014; Fig. S5, B and C). We also investigated late stages of autophagy, when autophagosomes fuse with lysosomes and their contents are degraded, and we found that JMY colocalizes with the late endosome and lysosome marker, Lamp1 (Fig. S5 D). Coutts and Lathangue (2015) previously reported that JMY does not colocalize with Lamp1, but we find the association most obvious when cells are treated with bafilomycin A to inhibit autolysosome maturation. These results indicate that JMY associates with autophagosomal membranes from nucleation to final fusion with lysosomal or endosomal membranes.

### The JMY-binding protein STRAP regulates autophagy

**Figure 3.**
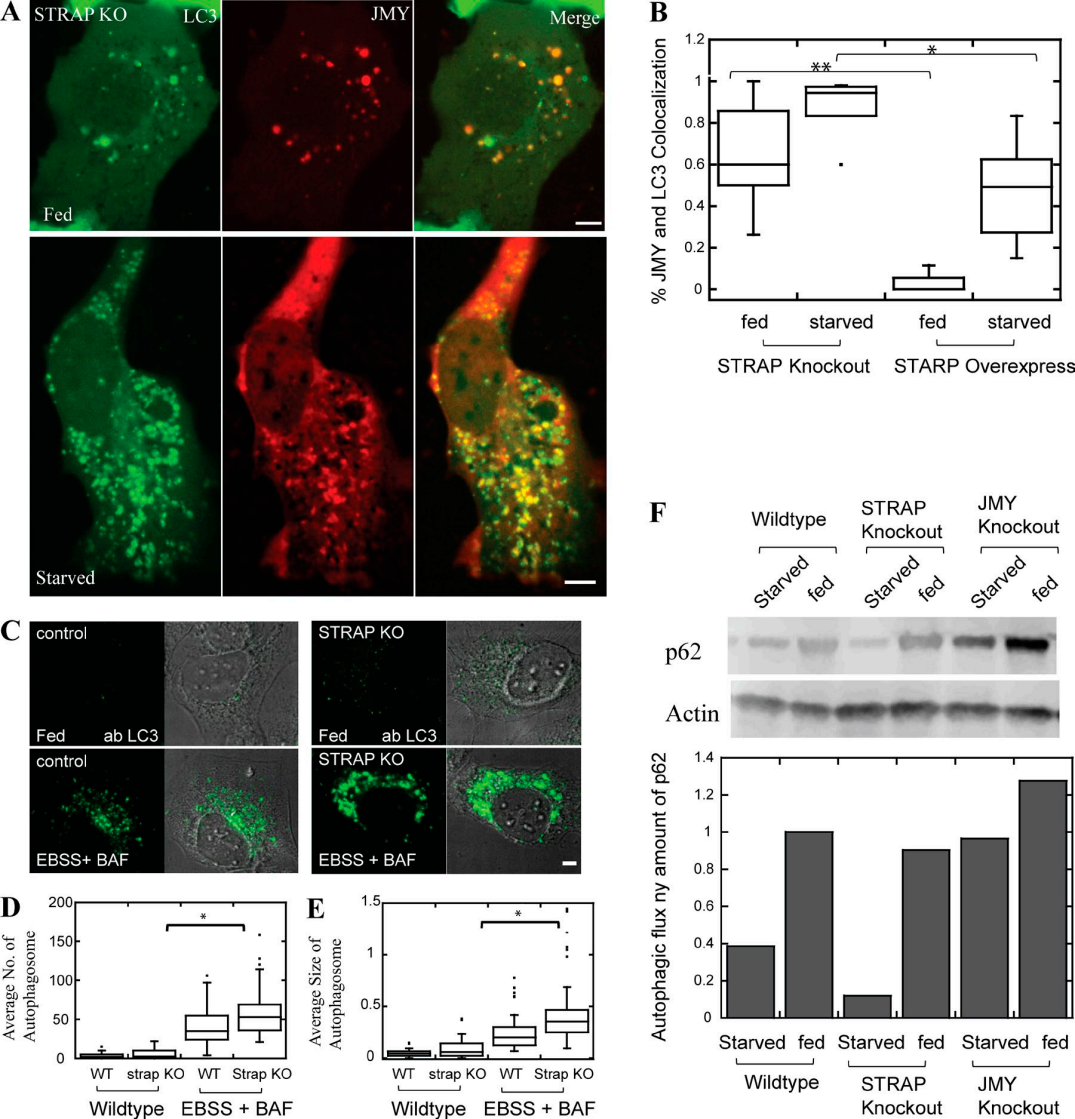
**STRAP is a negative regulator of autophagy. (A)** More JMY colocalizes with LC3 vesicles in STRAP knockout cell lines under both fed and starved conditions. **(B)** Quantification of colocalization of JMY and LC3 in cells either lacking STRAP or overexpressing STRAP (*n* = 3 independent experiments, 15 ~ 24 cells, **, P < 0.01; *, P < 0.05, Student’s *t* test). **(C)** Immunofluorescent staining of LC3 in WT and STRAP knockout cell lines. U2OS cells were either fed (top) or starved in EBSS with 100 nM Bafilomycin A to block autophagosome maturation (bottom). **(D and E)** Autophagosome number and size increase significantly in STRAP knockout cells (*n* = 3 independent experiments, 60 ∼ 68 cells, *, P < 0.05, Student’s *t* test). **(F)** Autophagic flux is increased in STRAP knockout cell lines, whereas it is decreased in JMY knockout cell lines. The autophagic flux is monitored by relative p62 protein concentrations measured by Western blot. Temperature for live cell imaging: 37°C. Scale bars: 5 µm.

STRAP binds directly to JMY, and JMY promotes autophagosome formation, so we wondered whether STRAP also regulates autophagy. To test this idea, we first overexpressed the STRAP protein in U2OS cells stably expressing GFP-LC3. We found that increased STRAP expression significantly reduces the average migration speed of LC3 vesicles (Fig. 2 G). We next quantified the degree of colocalization between JMY and LC3 in cells from which we had knocked out STRAP expression using CRISPR (Fig. S4). Loss of STRAP significantly increased the degree of colocalization between JMY and LC3 in both fed and starved cells (Fig. 3, A and B), suggesting that STRAP negatively regulates autophagosome movement, possibly by competing with LC3 for binding to JMY.

We next used our knockout cells to test whether STRAP functions as a negative regulator of autophagy. We used immunofluorescence to quantify LC3-positive autophagosomes in WT and STRAP knockout cells, and found that loss of STRAP expression significantly increased the number and size of endogenous autophagosomes under both fed and starved conditions (Fig. 3, C–E). We next tested whether loss of STRAP or JMY perturbs autophagic flux by quantifying cellular levels of p62, a cargo receptor and high-volume substrate for autophagy. Increased levels of p62 are associated with decreased autophagic flux and vice versa. In STRAP knockout cells, we detected decreased amounts of p62, consistent with an increase in autophagic flux. Conversely, JMY knockout cells contained higher steady-state concentrations of p62 compared with WT cells, indicating a decrease in autophagic flux (Fig. 3 F). Together, our results argue strongly that STRAP indeed functions as a negative regulator of autophagy.

### In vitro STRAP inhibits JMY’s intrinsic nucleation activity and its ability to activate the Arp2/3 complex

**Figure 4.**
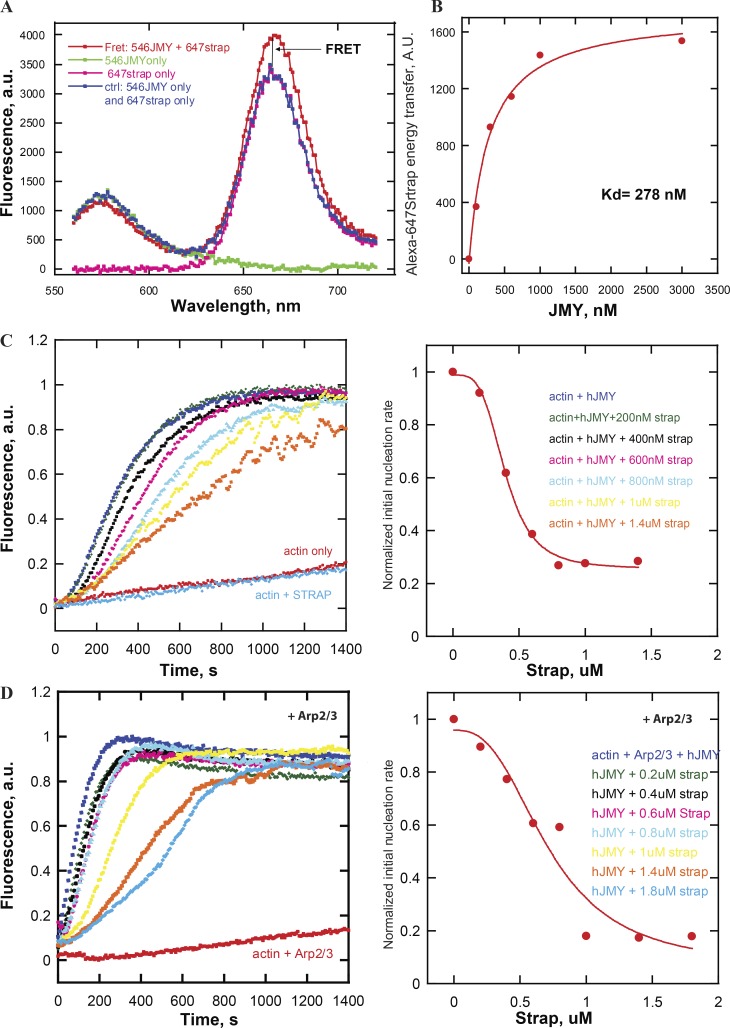
**In vitro purified STRAP binds JMY and inhibits both intrinsic nucleation activity and the ability to activate the Arp2/3 complex. (A)** FRET assay for the binding of purified STRAP to JMY. The curves are fluorescence intensity as a function of emission wavelength of 160 nM Alexa Fluor 546–labeled JMY (green) as donor, 600 nM Alexa Fluor 647–labeled STRAP (pink) as acceptor, and mixture of both fluorescently labeled proteins (red). The traces of donor alone and acceptor alone are combined to serve as baseline (blue), indicating no interaction. FRET is detected as an increase in acceptor emission intensity compared with baseline. **(B)** Binding isotherm for STRAP-JMY interaction derived from FRET assay (*K_d_* = ∼300 nM). Alexa Fluor 546–­labeled JMY is titrated in 300 nM Alexa Fluor 647–labeled STRAP in 20 mM Hepes buffer, pH 7.4, 100 mM KCl. **(C)** STRAP inhibits JMY’s intrinsic nucleation activity. Actin assembly was monitored by pyrene-actin fluorescence in various concentrations of STRAP (left). The initial slope (first 250 s) of each curve was normalized and plotted as a proxy for nucleation rate (right). **(D)** Dose dependence of STRAP inhibition of Arp2/3 complex activation by JMY. Pyrene-actin polymerization assay was performed in 50 mM KCl, 1 mM MgCl_2_, 1 mM EGTA, and 10 mM Imidazole, pH 7.0, buffer with 1 µM (5% labeled) actin, 200 nM JMY, and 25 nM Arp2/3 as noted and STRAP as indicated.

STRAP overexpression decreased motility of LC3-positive vesicles, so we used purified proteins to test whether STRAP directly regulates actin assembly by JMY. Using Forster resonance energy transfer (FRET) to detect interaction between donor- and acceptor-labeled proteins, we found that purified STRAP binds full-length JMY with submicromolar affinity (*K_d_* = 268 nM; Fig. 4, A and B). Remarkably, STRAP inhibits both the intrinsic actin nucleation activity of JMY (Fig. 4 C) and its ability to promote branched actin nucleation by the Arp2/3 complex (Fig. 4 D). In both sets of experiments, inhibition by STRAP was concentration-dependent, with half-maximal effective concentrations (EC_50_) approximately equal to the measured equilibrium dissociation constant (*K_d_*). Although STRAP’s inhibition of JMY was incomplete—plateauing at ∼80%—these experiments establish STRAP as the first known negative regulator of JMY’s actin assembly-promoting activities.

### LC3 binds JMY, enhances intrinsic actin nucleation activity, and promotes activation of the Arp2/3 complex

**Figure 5.**
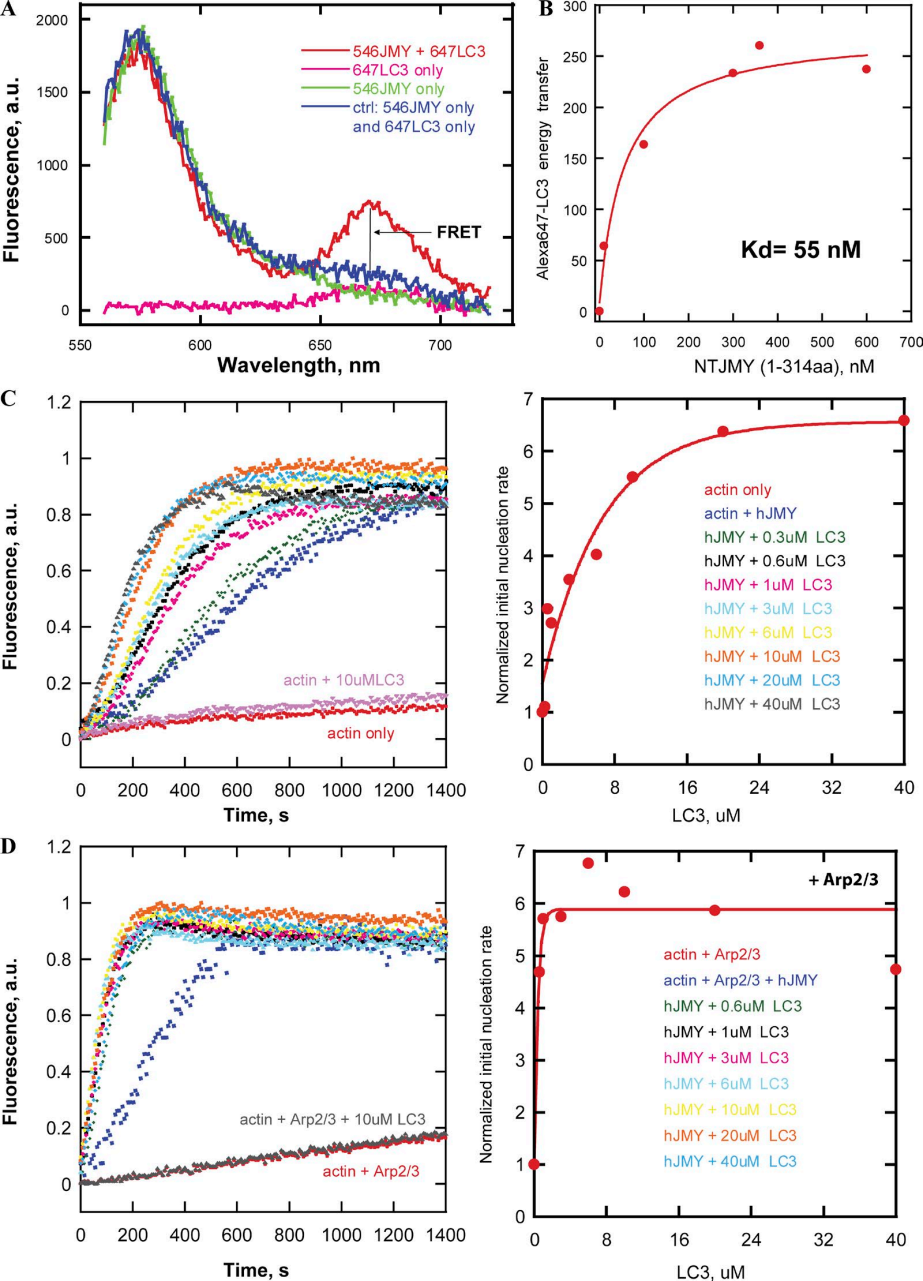
**In vitro LC3 enhances intrinsic nucleation and Arp2/3 activation by JMY. (A)** FRET assay for LC3 binding to JMY. The curves are fluorescence intensity as a function of emission wavelength of Alexa Fluor 546–labeled JMY (green) as donor, Alexa Fluor 647–labeled LC3 (pink) as acceptor, and mixture of both fluorescently labeled proteins (red). **(B)** Binding isotherm derived from FRET assay for the interaction of LC3 with the N-terminal region (residues 1–314) of JMY (*K_d_* = ∼50 nM). We added various concentrations of Alexa Fluor 546–labeled NT JMY to 200 nM Alexa Fluor 647–labeled LC3 in 100 mM KCl with 20 mM Hepes buffer, pH 7.4. **(C)** Dose dependence of LC3 stimulation of JMY’s intrinsic nucleation activity. LC3 promotes JMY’s intrinsic nucleation activity in pyrene-actin polymerization assay (left). The initial slope (first 250 s) of each curve is normalized and plotted as a proxy for nucleation rate (right). **(D)** Dose dependence of LC3 enhancement of Arp2/3 complex activation by JMY. Pyrene-actin polymerization assays were performed in 50 mM KCl, 1 mM MgCl_2_, 1 mM EGTA, and 10 mM Imidazole, pH 7.0, with 1 µM actin, 200 nM JMY, 25 nM Arp2/3 as noted, 16 mM NaCl, and LC3 as indicated.

Coutts and La Thangue (2015) reported that mutation of the LIR results in loss of JMY-associated actin structures in the cytoplasm. We also observed that vesicles containing both JMY and LC3 move through the cytoplasm on dynamic actin comet tails, so we hypothesized that LC3 might recruit JMY to membranes and stimulate its ability to make actin filaments. To test this idea, we first used a FRET-based binding assay to quantify the affinity of LC3 for full-length JMY and several JMY truncation mutants. We observed the highest affinity binding (*K_d_* = ∼55 nM) between LC3 and an N-terminal fragment of JMY spanning amino acids 1–314 (Fig. 5, A and B).

We next tested the effect of LC3 on JMY’s intrinsic and Arp2/3-dependent actin nucleation activities using pyrene-labeled actin. As judged by the time-dependent changes in pyrene fluorescence, soluble LC3 had no effect on actin assembly in the absence or presence of the Arp2/3 complex. In the presence of full-length JMY, however, LC3 enhanced the intrinsic actin nucleation activity in a concentration-dependent manner (Fig. 5 C). Similarly, LC3 accelerated actin assembly in the presence of both JMY and the Arp2/3 complex (Fig. 5 D).

**Figure 6.**
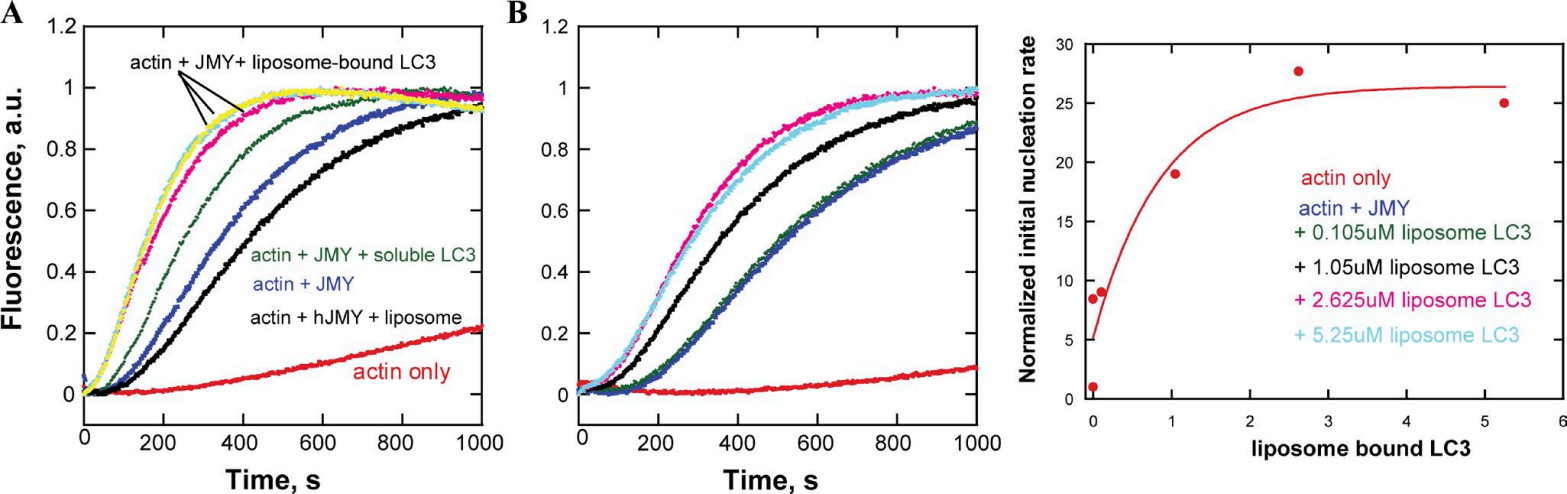
**Liposome-bound LC3 stimulates JMY’s nucleation activity. (A)** Liposome-bound LC3 promotes JMY’s intrinsic nucleation activity more potently than an equal amount of soluble JMY in a pyrene-actin polymerization assay. Time courses of actin assembly with liposome-bound LC3 (5.25 µM) are shown in three replicas (yellow, cyan, and pink) to confirm no error in pipetting of liposomes. **(B)** Dose-dependence of liposome-bound LC3 enhancement of JMY’s nucleation activity (middle). The initial slope (first 250 s) of each curve is normalized and plotted as a proxy for nucleation rate (right). Pyrene-actin polymerization assays were performed at 23°C in 50 mM KCl, 1 mM MgCl_2_, 1 mM EGTA, and 10 mM Imidazole, pH 7.0, with 1 µM actin, 200 nM JMY, 5.25 µM soluble LC3, and liposome-bound LC3 as indicated.

To test whether membrane-associated LC3 also enhances JMY’s nucleation activity, we made liposomes with a combination of phosphatidyl choline (PC) and phosphatidyl serine (PS), doped with 2% NiNTA-conjugated 1,2-dioleoyl-*sn*-glycero-3-succinyl (DGS). We included Ni-conjugated lipids to recruit His-tagged LC3 and made liposome-bound LC3. This liposome-bound LC3 stimulates JMY’s intrinsic and Arp2/3-dependent nucleation activities in pyrene-actin assembly assays even more robustly (Fig. 6, A and B) than soluble LC3.

### LC3 affects actin assembly via a cryptic regulatory sequence in the N-terminal region of JMY

We considered three possible mechanisms by which LC3 could enhance JMY’s ability to make actin filaments in both the absence and presence of the Apr2/3 complex. First, we investigated a role for LC3 oligomerization, in part because previous work demonstrated that dimerization of Spire-family nucleators (Quinlan et al., 2007) and WASP-family nucleation promoting factors (Padrick et al., 2011) significantly increases their activity. When we measured the molecular weight of purified LC3 in solution using sedimentation equilibrium ultracentrifugation, however, we found that, similar to a related protein, GABARAP (Coyle et al., 2002), LC3 is primarily monomeric at the concentrations used in our in vitro actin assembly experiments (*K_d_* for dimer formation >700 μM). For this reason, LC3 oligomerization is unlikely to explain the enhancement of JMY activity. Second, we wondered whether JMY has a cryptic LIR buried somewhere within its actin regulatory sequence (PWWWCA). This hypothesis is appealing because intra- and intermolecular contacts with Arp2/3-activating VCA sequences regulate the activity of other nucleation promoting factors (e.g., WASP and WAVE). In a FRET-based binding assay, LC3 did not interact with the C-terminal fragment of JMY (CCPWWWCA, amino acids 315–983) that contains the coiled-coil, proline-rich, and WWWCA domains (Fig. S6 B). In addition, we found that LC3 had no effect on actin nucleation or Arp2/3 activation by C-terminal JMY constructs (Fig. S6 A). These results fit with the previous identification of a single LIR near JMY’s N terminus (Coutts and La Thangue, 2015).

**Figure 7.**
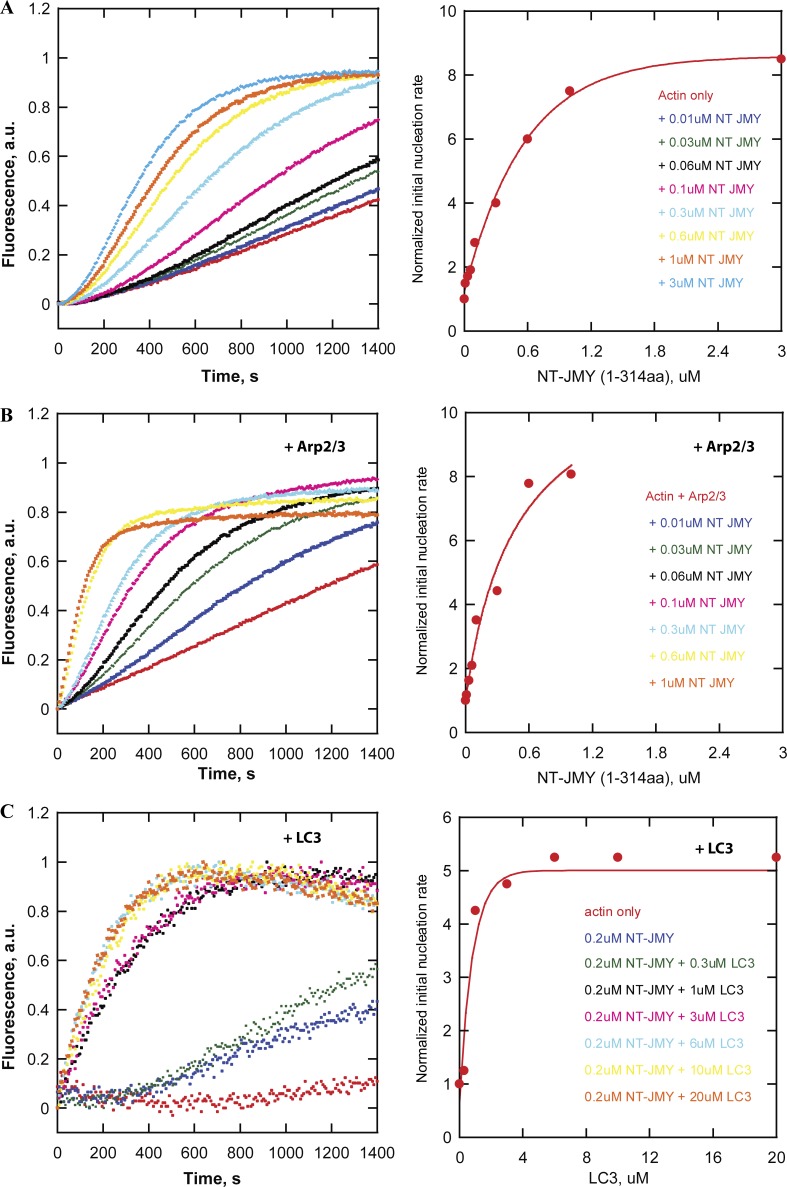
**The effect of LC3 on actin nucleation and Arp2/3 activation by JMY reveals a cryptic actin regulatory sequence in the N-terminal region of JMY. (A and B)** NT JMY (residues 1–314) promotes concentration-dependent actin nucleation in the absence (A) and presence (B) of the Arp2/3 complex. Actin assembly was monitored by the fluorescence of pyrene-labeled actin (left). The initial slope of each curve is normalized and plotted as a proxy for nucleation rate (right). Pyrene-actin polymerization assays are performed at 23°C in 50 mM KCl, 1 mM MgCl_2_, 1 mM EGTA, and 10 mM Imidazole, pH 7.0, with 2 µM actin, 25 nM Arp2/3, and NT JMY as noted. **(C)** LC3 enhances NT JMY (residues 1–314) intrinsic actin nucleation activity. Actin assembly in the presence of various concentrations of LC3 was monitored by the fluorescence of pyrene-labeled actin (left). The initial slope (first 250 s) of each curve is normalized and plotted as a proxy for nucleation rate (right). Buffer conditions: same as in A with 1 µM actin, 200 nM NT JMY, 16 mM NaCl, and LC3 as indicated.

Finally, we asked whether LC3 regulates nucleation activity via interaction with JMY’s N-terminal LIR. We began by testing whether JMY’s activities are inhibited by an intramolecular interaction, similar to those that regulate some formins (Li and Higgs, 2003) and WASP-family nucleation promoting factors (Rohatgi et al., 1999). Briefly, we tested whether truncation mutants containing the N-terminal region of JMY (NT-JMY, amino acids 1–314) can inhibit actin assembly stimulated by the C-terminal region (CCPWWWCA, amino acids 315–983). To our surprise, addition of the NT-JMY truncation mutant actually increases the rate of actin nucleation by the CCPWWWCA construct (Fig. S6 D). This increased activity turns out to reflect the contribution of a cryptic actin-nucleating region within the NT-JMY construct. Although this region contains no identifiable actin-binding motifs, we found that the NT-JMY truncation mutant by itself accelerates actin assembly in a concentration-dependent manner (Fig. 7, A and B). Moreover, addition of LC3 further enhances actin nucleation by NT-JMY (Fig. 7 C). This previously unsuspected nucleation activity suggests that NT-JMY contains one or more cryptic actin binding sites, which we confirmed by measuring FRET between NT-JMY and fluorescent actin monomers bound to the polymerization inhibitor latrunculin-B (Fig. S6 E). We conclude, therefore, that LC3 promotes JMY-dependent actin nucleation by interacting with, and enhancing the activity of, a previously unknown actin regulatory sequence in the N-terminal region.

### In vitro reconstitution of LC3- and JMY-dependent actin comet tail formation from purified components

**Figure 8.**
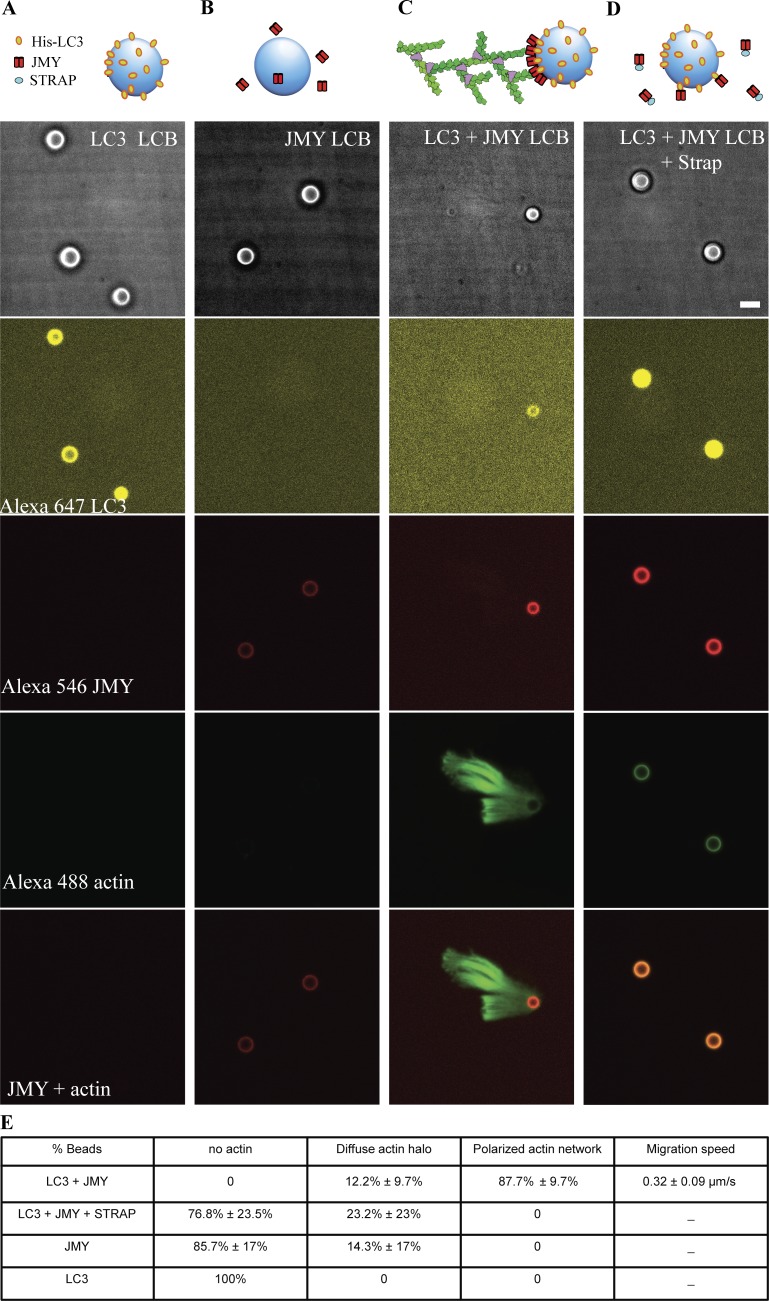
**Reconstitution of LC3- and JMY-dependent actin comet tail formation from purified components.** We mixed lipid-coated 4.5-µm glass microspheres with his-tagged LC3 (A); full-length JMY (B); both his-tagged LC3 and JMY (C); and STRAP together with his-tagged LC3 and JMY (D). From top to bottom: bright-field images; Alexa Fluor 647–labeled LC3; Alexa Fluor 546–labeled JMY; Alexa Fluor 488–labeled actin; merger of fluorescent signal from Alexa Fluor 546–labeled JMY and Alexa Fluor 488–labeled actin. **(E)** Quantification of actin network on lipid-coated bead surface. Buffer conditions: 8 µM actin (10% labeled with Alexa Fluor 488), 200 nM Arp2/3, 400 nM Capping protein, 8 µM profilin, 2 µM STRAP as noted, 1 mg/ml BSA, 1 mg/ml β-casein, 50 mM KCl, 1 mM MgCl_2_, 1 mM EGTA, 0.2 mM ATP, and 20 mM Hepes, pH 7.0. Temperature: 23°C. Scale bars, 5 µm.

Finally, we asked whether LC3 is sufficient to recruit JMY to autophagosome membranes and stimulate actin-based motility by reconstituting the process in vitro, using purified components. We coated 4.5-µm glass microspheres with a combination of PC and PS, doped with 2% NiNTA-conjugated DGS. We included Ni-conjugated lipids to recruit His-tagged LC3 that was labeled with a fluorescent dye, Alexa Fluor 647 (Fig. 8 A). By themselves, NiNTA-doped, lipid-coated microspheres did not recruit full-length JMY, labeled with Alexa Fluor 546 (Fig. 8 B). The LC3-bound lipid-coated beads did, however, recruit full-length JMY and—in the presence of actin, capping protein, profilin, and the Arp2/3 complex—initiated the assembly of polarized, branched actin networks (Fig. 8 C) similar to the motile actin comet tails we observed in live cells. Interestingly, addition of soluble STRAP to this reaction does not prevent recruitment of JMY to the LC3-coated microspheres, but strongly suppresses formation of a branched actin network (Fig. 8 D), consistent with STRAP’s ability to inhibit JMY’s actin-nucleating activity and with an inhibitory role for STRAP in JMY-mediated autophagosome movement.

## Discussion

Although it promotes actin filament assembly by multiple mechanisms (Zuchero et al., 2009), JMY was first described as a coactivator of p53-mediated apoptosis that accumulates in the nucleus in response to DNA damage. Early work identified several binding partners, including p300 and STRAP, that collaborate with JMY to promote apoptosis, but the regulation of JMY-mediated actin assembly has remained mysterious. Here we find that JMY’s nuclear partner, STRAP, also modulates its ability to create actin filaments in the cytoplasm. Previous work showed that DNA damage induces ATM-dependent phosphorylation of STRAP, leading to its nuclear localization and the formation of a complex between STRAP, JMY, and p300 (Demonacos et al., 2001). This is believed to be part of the reason JMY accumulates in the nucleus after DNA damage. We find, however, that in the absence of DNA damage, STRAP also colocalizes with JMY in the cytoplasm on nonmotile vesicles; and further that STRAP suppresses JMY’s actin nucleation activities in vitro. One consequence of this inhibition is that JMY likely cannot promote actin assembly when bound to STRAP inside the nucleus.

The best understood nucleation promoting factors are regulated by autoinhibitory interactions within a single molecule (e.g., N-WASP) or a regulatory complex (e.g., the wave regulatory complex). Upstream signaling molecules stimulate the activity of these NPFs by disruption of autoinhibition, either allosterically (Rohatgi et al., 2000; Padrick et al., 2008; Koronakis et al., 2011) or competitively (Okrut et al., 2015). The ability of LC3 to promote actin nucleation by JMY, however, does not involve disrupting an autoinhibitory interaction, but rather the enhancement of a previously unsuspected nucleation activity associated with JMY’s N-terminal LIR sequence. Although surprising, this result explains a previous report that the N terminal region of JMY is essential for its nucleation activity in the cytoplasm (Coutts and La Thangue, 2015).

We cannot rule out the existence of additional regulatory molecules, but based on our results we suggest a two-state or two-compartment model for JMY regulation. In this model, regulation consists of shuttling JMY molecules from a negative regulator on one membrane compartment to a positive regulator on a different set of membranes. Specifically, when cells are in the normal, fed state, JMY is sequestered and held in an inactive state by interacting with STRAP. During starvation-induced autophagy, LC3 titrates JMY away from STRAP and onto autophagosomal membranes, where its nucleation activity generates branched actin networks. Whether this shuttling involves formation of transient ternary complexes between STRAP, JMY, and LC3 or requires other proteins to mediate the transfer is a subject for further study.

Given that JMY and WHAMM both promote actin comet tail formation on autophagosomal membranes, why do mammalian cells require both nucleation promoting factors for efficient autophagy? A simple hypothesis is that the activities of WHAMM and JMY are required at different times and/or places. Autophagosome formation proceeds via multiple morphologically and biochemically distinct steps: (a) localized initiation of a phagophore, (b) nucleation and (c) expansion of the phagophore membrane, and (d) closure and (e) detachment of the autophagosome from the initiation site (Graef et al., 2013). Once detached, autophagosomes must move to and fuse with lysosomes/late endosomes. WHAMM is recruited to membranes by an early event in phagophore initiation: phosphorylation of PI in the ER to form PI(3)P. This acidic phospholipid binds and recruits WHAMM via interaction with PX domain–like sequences in the N-terminal region of the protein (Mathiowetz et al., 2017). In contrast, JMY interacts with LC3, whose lipidation happens after WHAMM is recruited to PI3P sites (Mathiowetz et al., 2017). This might suggest that JMY is strictly downstream of WHAMM, but the story is not so simple because we also observe colocalization and comigration of JMY with proteins involved in the initiation and nucleation phases of phagophore assembly (DFCP1 and ATG9; Fig. S5, B and C). These observations make it difficult to establish a clear difference in the timing of JMY and WHAMM recruitment during early stages of autophagosome formation. The fact that JMY, but not WHAMM, colocalizes with Lamp1 suggests that JMY activity might play a unique role in some late phases of autophagy.

The direct regulation of its actin nucleation activity by LC3 suggests that JMY plays a particularly important role in LC3-associated autophagic events, such as expansion of phagophore membranes; detachment of autophagosomes from sites of biogenesis; and fusion with lysosomes. This idea is consistent with the fact that JMY knockout mimics the effect of inhibiting the Arp2/3 complex, decreasing accumulation of autophagosomes in the perinuclear region when autophagic flux is blocked by Bafilomycin A (Fig. S4, D–F). This phenotype is consistent with failure to detach autophagosomes from parental membrane compartments and/or their inability to move and fuse with downstream compartments.

## Materials and methods

### Constructs and reagents

Human JMY and mouse STRAP (also named TTC5) derivatives were cloned into pHR vector with a C-terminal mGFP, mCherry, or SNAP tag for mammalian expression. Human JMY, full-length and truncations, were cloned into pFastBac with an N-terminal GST tag, followed by a prescission protease cleavage site and a SNAP tag for future labeling. Mouse STRAP, full length, was cloned into pet20b vector with a C-terminal 6xHis tag and an N-terminal lysine-cysteine-lysine (KCK) tag for future labeling. Human EGFP-LC3B was purchased from Addgene (11546) for mammalian expression, and human LC3 was cloned into pet20b vector with a C-terminal 6xHis tag and an N-terminal KCK tag for future labeling. EGFP-DFCP1, GFP-ATG2, GFP-ATG9, and GFP-VAPA were purchased from Addgene. EGFP-F-Tractin, mTagBFP2-lifeact, and mCherry-Lamp1 were purchased from Davidson Lab Plasmid of UCSF imaging center.

### Cell lines, lentiviral infection, and transfection

U2OS (ATCC) cells were cultured in DMEM supplemented with 10% FBS, 2 mM l-glutamine, and penicillin-streptomycin (Thermo Fisher Scientific). Lentiviral JMY-mGFP, JMY-mCherry, and STRAP-mCherry were made from HEK293 cells and infected U2OS cells. A U2OS cell line that stably expresses EGFP-LC3B was made by G418 screening and FACs sorting. Other mammalian expression constructs were transiently transfected into U2OS cells by using Lipofectamine 3000 (Invitrogen).

### CRISPR knockout cell lines

The CRISPR knockout cell line of JMY was made according to the protocol described in Ran et al. (2013). The knockout efficiency of JMY in single-cell colonies was validated by real-time RT-PCR, Western blot, and immunofluorescence by using a home-made rabbit polyclonal antibody against hJMY. The single guide RNA (sgRNA) for JMY knockout is 5′-TCGCGCTCGTCGAACACATGGGG-3′. The sgRNA used for STRAP knockout is 5′-CTTTGACTTGCATGCTCAACAGG-3′. The knockout efficiency was confirmed by real-time RT-PCR (Bio-Rad).

### Live-cell imaging

Microscopy was performed on an inverted microscope (Nikon Ti-E) equipped with a spinning-disk confocal system (Spectral Diskovery) and imaged with a 60× Apo TIRF Objective (NA 1.49) and EMCCD camera (Andor iXon Ultra). For live unstarved cell studies, images were acquired at 37°C with 5% CO_2_ in an Okolab stagetop incubator. Starved cells were imaged in HBSS (Sigma) supplemented with 20 mM Hepes and 100 nM Bafilomycin A. Images were captured at 1.0-s intervals at 16-bit resolution using Micro-manager software. Videos and images were prepared using Fiji software (National Institutes of Health). Migration speeds and diameters were all determined using the manual tracking or manual measurement features of Fiji. To avoid observer bias, we acquired and analyzed images double-blind.

### Protein expression, purification, and labeling

Sf9 cells were cultured in insect medium (Lonza Biowhittakar) and were infected by baculovirus of human GST-SNAP-JMY full length, GST-SNAP-JMY (1–314), and GST-SNAP-JMY (863–983). Insect cells were harvested 72 h after infection, freshly lysed, and purified. Using a microfluidizer, cells were lysed in PBS, 1 mM EDTA, 10 mM β-mercaptoethanol, and 1 mM PMSF. The lysates were spun at 25,000 rpm (Beckman Ultracentrifuge, Ti45 rotor) for 20 min, and supernatants were batch bound to glutathione Sepharose 4b (GE Healthcare). Resin was washed with lysis buffer containing 0.1% Triton X-100 and eluted with freshly made 33-mM reduced glutathione in 50 mM Tris, pH 8.0. GST-SNAP-JMY was dialyzed and cleaved by GST-tagged prescission protease for 3 h and rebound to glutathione Sepharose 4b. The unbound fractions were collected and concentrated by dry sucrose and further purified by a gel filtration Superdex 200 column, followed by an anion exchange column MonoQ (GE Healthcare). The pure untagged SNAP-JMY was dialyzed into storage buffer (20 mM Hepes, 100 mM KCl, 1 mM EDTA, 0.5 mM Tris(2-carboxyethyl)phosphine [TCEP], and 20% glycerol, pH 7.4) and snap-frozen in liquid nitrogen. SNAP-JMY and derivatives were labeled with SNAP-cell-TMR-star (New England Lab) following the manufacturer’s protocol, and soluble free dye was removed with a G25 Sephadex column.

Expression of JMY-CCPWWWCA (315-983)-TEV-his6 construct was performed in BL21 Rosetta *Escherichia coli*, induced with 30 µM IPTG at 18°C for 16 h. Using a microfluidizer, bacteria were lysed into 50 mM NaH_2_PO_4_, 300 mM NaCl, 10 mM imidazole, 10 mM β-mercaptoethanol, and 1 mM PMSF, pH 8. High-speed supernatant was then batch bound to Ni-NTA resin (Qiagen). Resin was washed with lysis buffer containing 20 mM imidazole and eluted with lysis buffer containing 200 mM imidazole. JMY-CCPWWWCA (315–983)-TEV-his6 was then dialyzed and cleaved by TEV protease for 3 h, and was rebound to Ni-NTA. The unbound fractions were collected and further purified with a cation exchange MonoS column (GE Healthcare). Pure JMY-CCPWWWCA were dialyzed into storage buffer (PBS, 1 mM EDTA, 0.5 mM TCEP, and 30% glycerol) and snap-frozen with liquid nitrogen before −80°C storage.

Expression of KCK-mSTRAP-his6 construct was performed in BL21 Rosetta *E. coli*, induced with 200 µM IPTG at 18°C for 16 h. Using a microfluidizer, bacteria were lysed into 50 mM NaH_2_PO_4_, 300 mM NaCl, 10 mM imidazole, 10 mM β-mercaptoethanol, and 1 mM PMSF, pH 8. High-speed supernatant was then batch bound to Ni-NTA resin (Qiagen). Resin was washed with lysis buffer containing 20 mM imidazole and eluted with lysis buffer containing 200 mM imidazole. mSTRAP was dialyzed into 10 mM Hepes, pH 6.5, 1 mM EDTA, and 1 mM DTT. Proteins were further purified with a cation exchange MonoS column. Pure mSTRAP were dialyzed into storage buffer (20 mM Hepes and 0.5 mM TCEP, pH 7.4) and frozen with liquid nitrogen before −80°C storage.

Expression of hLC3-KCK-his6 was performed in BL21 Rosetta *E. coli*, induced with 50 µM IPTG at 18°C for 16 h. Using a microfluidizer, bacteria were lysed into PBS buffer, 10 mM β-mercaptoethanol, and 1 mM PMSF. High-speed supernatant was then batch bound to Ni-NTA resin (QIAGEN). Resin was washed with lysis buffer containing 20 mM imidazole and eluted with lysis buffer containing 200 mM imidazole. hLC3 was then concentrated with 3KD Amico Ultra centrifugal filters (Millipore). Proteins were further purified with a Superdex 200 gel filtration column (GE Healthcare). Pure mSTRAP were liquid nitrogen snap-frozen in storage buffer (20 mM Hepes, 50 mM NaCl, and 0.5 mM TCEP, pH 7.4) and stored at −80°C.

Labeling was achieved by combining reduced KCK-mSTRAP or LC3-KCK with 5 molar excess Alexa Fluor 546–maleimide (GE Healthcare) on ice for 15 min before quenching with 10 mM DTT. Soluble free dye was removed with a G25 Sephadex column. Labeling efficiency was assessed with a spectrophotometer.

Cytoplasmic actin was purified from rabbit skeleton muscle based on the methods described in Hu and Kuhn (2012). Gel-filtered monomeric actin was stored in buffer containing 2 mM Tris, pH 8.0, 0.5 mM TCEP, 0.1 mM CaCl_2_, and 0.2 mM ATP. Actin was labeled on Cys-374 with Alexa Fluor 488–maleimide (Invitrogen) using the same method used for labeling KCK-mSTRAP. Arp2/3 complex was purified from *Acanthamoeba castellanii* following the methods described in Dayel et al. (2001). Human profilin I was purified using established protocols (Kaiser et al., 1989). Recombinant mouse capping protein was purified using a protocol adapted from Palmgren et al. (2001).

### Actin polymerization assays

Actin was labeled with pyrene iodoacetamide as described previously (Cooper et al., 1983) and stored on ice. For all assays, Arp2/3 was thawed daily and diluted with 1 mg/ml BSA in buffer A (0.2 mM ATP, 0.5 mM TCEP, 0.1 mM CaCl_2_, 0.02% wt/vol sodium azide, and 2 mM Tris-HCl, pH 8.0 at 4°C). Actin polymerization assays were performed in 1× KMEI (50 mM KCl, 1 mM MgCl_2_, 1 mM EGTA, and 10 mM imidazole, pH 7.0). Ca^2+^-actin was converted into Mg^2+^-actin by incubating actin in 50 mM MgCl_2_ and 0.2 mM EGTA for 2 min before adding 10× KMEI and test components. Pyrene fluorescence was measured with a Snergy4 plate reader (BioTek). Unless otherwise noted, polymerization reactions contained 1 µM actin (labeled with 5% pyrene), 25 nM Arp2/3, and 200 nM JMY. To normalize fluorimetry data, we subtracted the offset from zero and then divided the plateau value of actin polymerization. For initial nucleation rate analysis, we fitted the first 100 s (in the presence of Arp2/3) or 250 s (in the absence of Arp2/3) of the kinetic data into a linear plot and normalized the slope by dividing the slope of actin only or actin plus Arp2/3.

### FRET

We first scanned the donor and the acceptor separately, and the corresponding curves of donor and acceptor were added to serve as baseline indicating no interaction. We then mixed the donor and acceptor, and FRET was indicated by the increase in acceptor emission intensity compared with the baseline. mSTRAP or LC3 (donor) labeled with Alexa Fluor 546–maleimide was titrated into 140 nM JMY (acceptor) labeled with Alexa Fluor 647–SNAP in buffer with 20 mM Hepes and 100 mM NaCl, pH 7.4. The FRET experiment was performed in a Synergy 4 platereader (BioTek) and a ISS PCI/K2 fluorimeter.

### Liposome and lipid-coated bead preparation

To make lipid-coated beads, glass beads (diameter 4.5 µm; Bangs Technology) was first cleaned with 1 M HCl, 5 M NaOH, 1 mM EGTA, 70% ethanol, and pure ethanol in sequence and washed with MilliQ water between steps. The small unilamellar vesicles (SUVs) were made by mixing 7.5 µmol PC (l-α-phosphatidylcholine), 2.3 µmol PS (l-α-phosphatidylserine), and 0.2 µmol Ni-NTA-DGS (18:1 DGS-NTA [Ni+2] 1,2-dioleoyl-sn-glycero-3-[(*N*-(5-amino-1-carboxypentyl)iminodiacetic acid)succinyl]), which was frozen and thawed 20 times and spun at 21,000 *g* for 30 min. The top 80% of supernatant was removed, and the tube was filled with nitrogen gas. These SUV vesicles were further extruded through 100-nm pore size filters to make liposome. Lipid-coated beads were made by mixing 7.5 µl of 10% bead slurry and 12.5 µl of 4 µM SUV, bath-sonicating with rotation for 15 min, and then washing five times with MilliQ water. The lipid-coated beads were stored in 20 mM Hepes, pH 7.4, and 150 mM NaCl for up to a week.

### Reconstitution of actin-based bead motility

To load proteins to lipid-coated beads, we mixed 4 µM of 1% Alexa Fluor 647–labeled LC3-His6 and 4 µM of 1% Alexa Fluor 546–labeled SNAP-JMY with rotation in a cold room for 1 h. Then proteins were mixed with 20 µl lipid-coated beads in buffer including 1 mg/ml BSA, 1 mg/ml β-casein, 20 mM Hepes, pH 7.0, 100 mM KCl, and 0.5 mM TCEP and rotated for 10 min. Unbound proteins were removed by low-speed centrifugation (200 *g*) five times.

Labeled and unlabeled Ca-ATP actin was diluted to the desired labeled fraction, mixed 9:1 with 10× magnesium exchange buffer (10× ME = 10 mM EGTA and 1 mM MgCl_2_), and incubated on ice for 2 min to form 4× final concentrations (4 × 8 µM) of Mg-ATP actin. 8 µl Mg-ATP actin was placed at the bottom of a 1.5-ml Eppendorf tube, and 7 µl of motility protein mixtures (200 nM Arp2/3, 400 nM CP, and 8 µM profilin) were added, along with 1 µl coated beads on the side of the tube. The two drops were washed together with 16 µl 2× buffer (2× = 100 mM KCl, 2 mM MgCl_2_, 2 mM EGTA, 20 mM imidazole, pH 7.0, 200 mM DTT, 0.4 mM ATP, 30 mM glucose, 0.5% 1500 cP methylcellulose, 40 mg/ml catalase, and 200 µg/ml glucose oxidase), and the reaction mixture was placed in either a flow cell or slide-coverslip blocked in 1 mg/ml BSA.

### Online supplemental material

Fig. S1 shows quantification of colocalization of JMY, LC3, and STRAP in cells expressing three proteins and a subset of JMY- and STRAP-positive puncta enclosed by LC3 vesicle. Fig. S2 shows LC3- and JMY-positive puncta colocalized on motile vesicles in U2OS cells expressing LC3 and JMY, whereas JMY and STRAP positive puncta are nonmotile in cells expressing JMY and STRAP. Fig. S3 shows that JMY comigrates with ER-resident protein VAPA in an actin-propelled manner. Fig. S4 shows a test of JMY or STRAP knockout cell lines, total purified proteins used, and JMY-LC3–positive vesicles that lost perinuclear enrichment when the actin branched network was inhibited. Fig. S5 shows wortmannin A treatment of cells and JMY colocalized with autophagy markers at different stages. Fig. S6 shows biochemical analysis of JMY and related proteins. Video 1 shows JMY and STRAP colocalized on nonmotile puncta in a fed cell. Upon starvation, more JMY colocalized and comigrated with LC3. Video 2 shows that JMY- and LC3-positive puncta partially colocalize and comigrate. Video 3 shows that JMY- and STRAP-positive puncta partially colocalize and are nonmotile. Video 4 shows that polarized actin network propels JMY puncta movement. Video 5 shows that polarized actin network propels JMY and LC3 double positive puncta movement. Video 6 shows that CK666 inhibits JMY and LC3 double positive puncta movement. Video 7 shows that LC3 vesicles are much less motile in the JMY knockout cell line.

## Supplementary Material

Supplemental Material (PDF)

Video 1

Video 2

Video 3

Video 4

Video 5

Video 6

Video 7
