# Supplementary material for: LC3 and STRAP regulate actin filament assembly by JMY during autophagosome formation
Source: J Cell Biol. 2019 Jan 7;218(1):251–66. doi: 10.1083/jcb.201802157 (PMC6314544; doi:10.1083/jcb.201802157)
Supplement: Supplemental Material (PDF) [file JCB_201802157_sm.pdf]

Supplemental material

Hu and Mullins, https://doi.org/10.1083/jcb.201802157

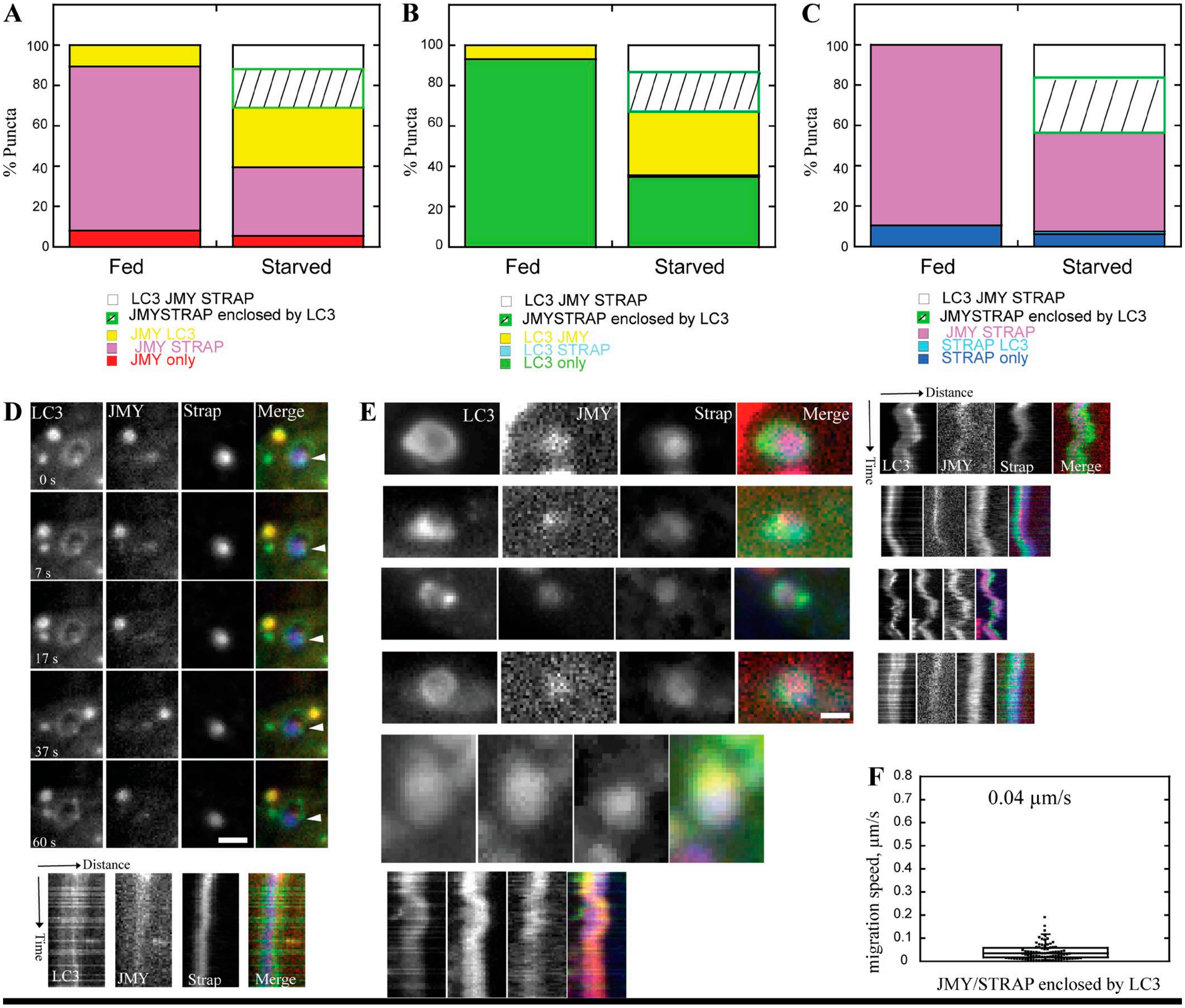

Figure S1. **Colocalization of JMY, LC3, and STRAP. (A–C)** Quantification of colocalization of JMY, LC3, and STRAP in cells expressing three proteins. The percentage is normalized to total number of JMY per cell (A), LC3 (B), and STRAP (C; *n* = 3 independent experiments). **(D)** A subset of JMY- and STRAP-positive puncta (arrowhead) is enclosed by LC3 vesicle. **(E)** More examples show that LC3 vesicles enclose JMY- and STRAP-positive puncta. **(F)** Quantification of migration speed. Scale bars: 2 µm.

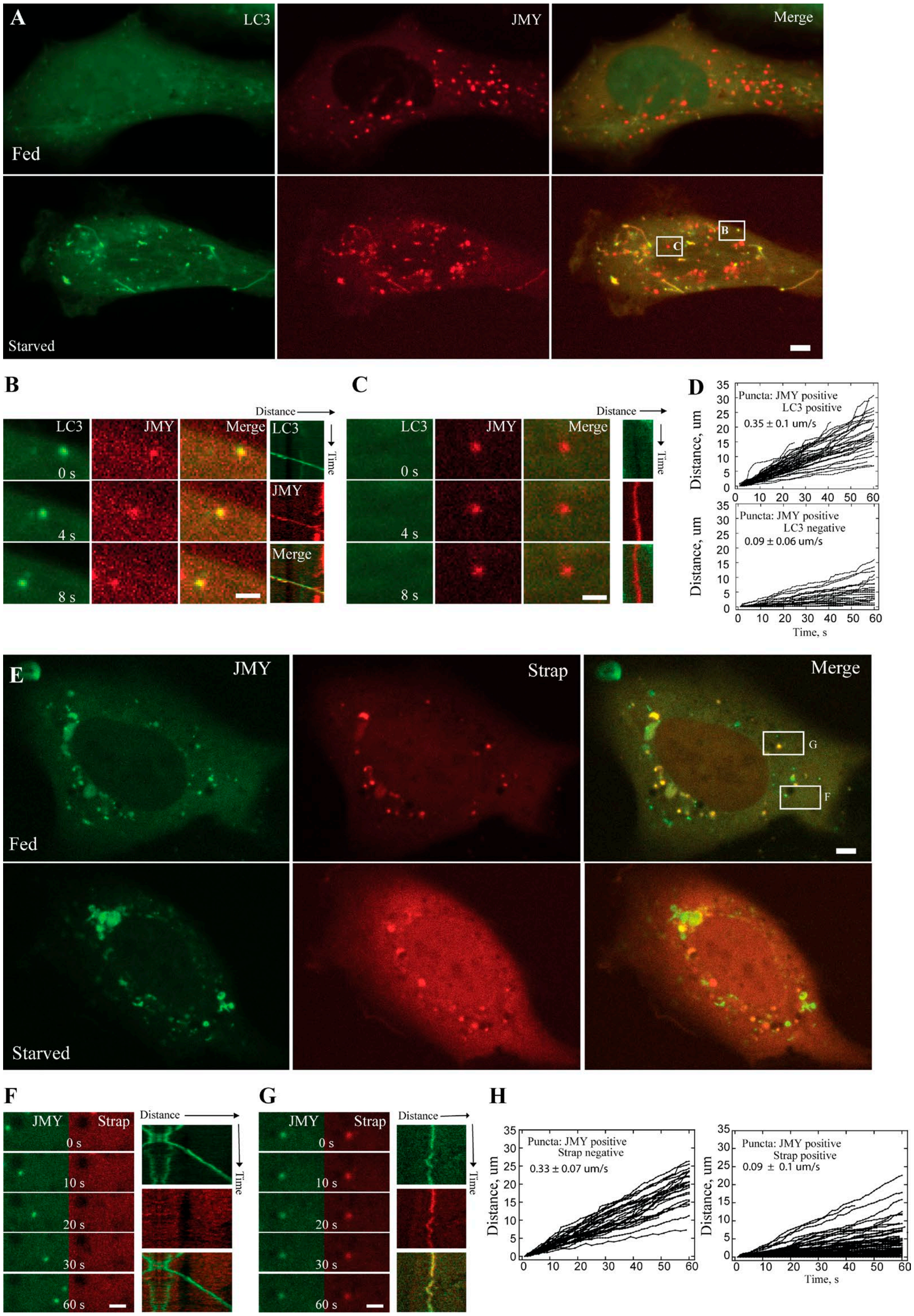

Figure S2. **LC3- and JMY-positive puncta colocalize on motile vesicles in U2OS cells expressing LC3 and JMY, whereas JMY- and STRAP-positive puncta are nonmotile in cells expressing JMY and STRAP. (A)** GFP-LC3B and JMY-mCherry partially colocalize upon starvation-induced (HBSS) autophagy (bottom). **(B)** Punctate structures positive for both JMY and LC3 are highly motile. **(C)** Puncta that contain only JMY and lack LC3 are nonmotile. **(D)** Raw distance versus time plots of JMY-only and JMY/LC3-positive foci (*n* = 3 independent experiments, 11 cells). **(E)** Partial colocalization of JMY and STRAP to cytoplasmic foci. **(F)** JMY puncta that do not contain STRAP are motile. **(G)** JMY puncta that also contain STRAP are nonmotile. **(H)** Raw distance versus time plots of JMY-positive foci in JMY- and STRAP-coexpressing cells (*n* = 3 independent experiments, eight cells). Scale bars: whole cell, 5 µm; zoom-in box, 2 µm.

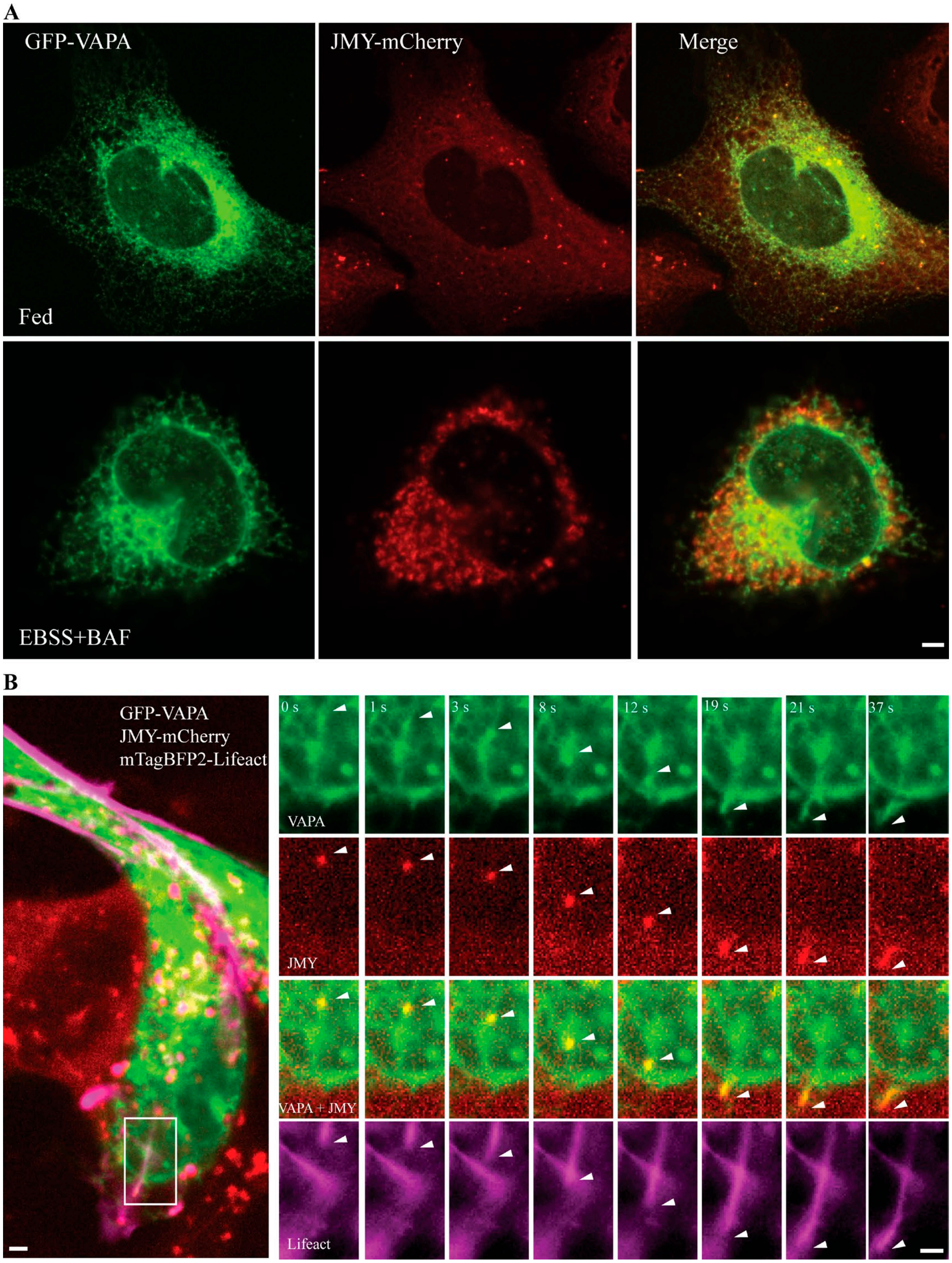

Figure S3. **JMY comigrates with ER-resident protein VAPA in an actin-propelled manner. (A)** JMY partially colocalizes with VAPA in immunofluorescent staining. **(B)** Actin-propelled JMY vesicles colocalize and comigrate with ER-resident protein VAPA. JMY vesicles (red) coupled with actin comet tails (magenta) travel along VAP-A–labeled (green) ER tubule. Arrowheads in all panels denote the position of the JMY vesicle. Scale bars: whole cell, 5 µm; zoom-in box, 2 µm.

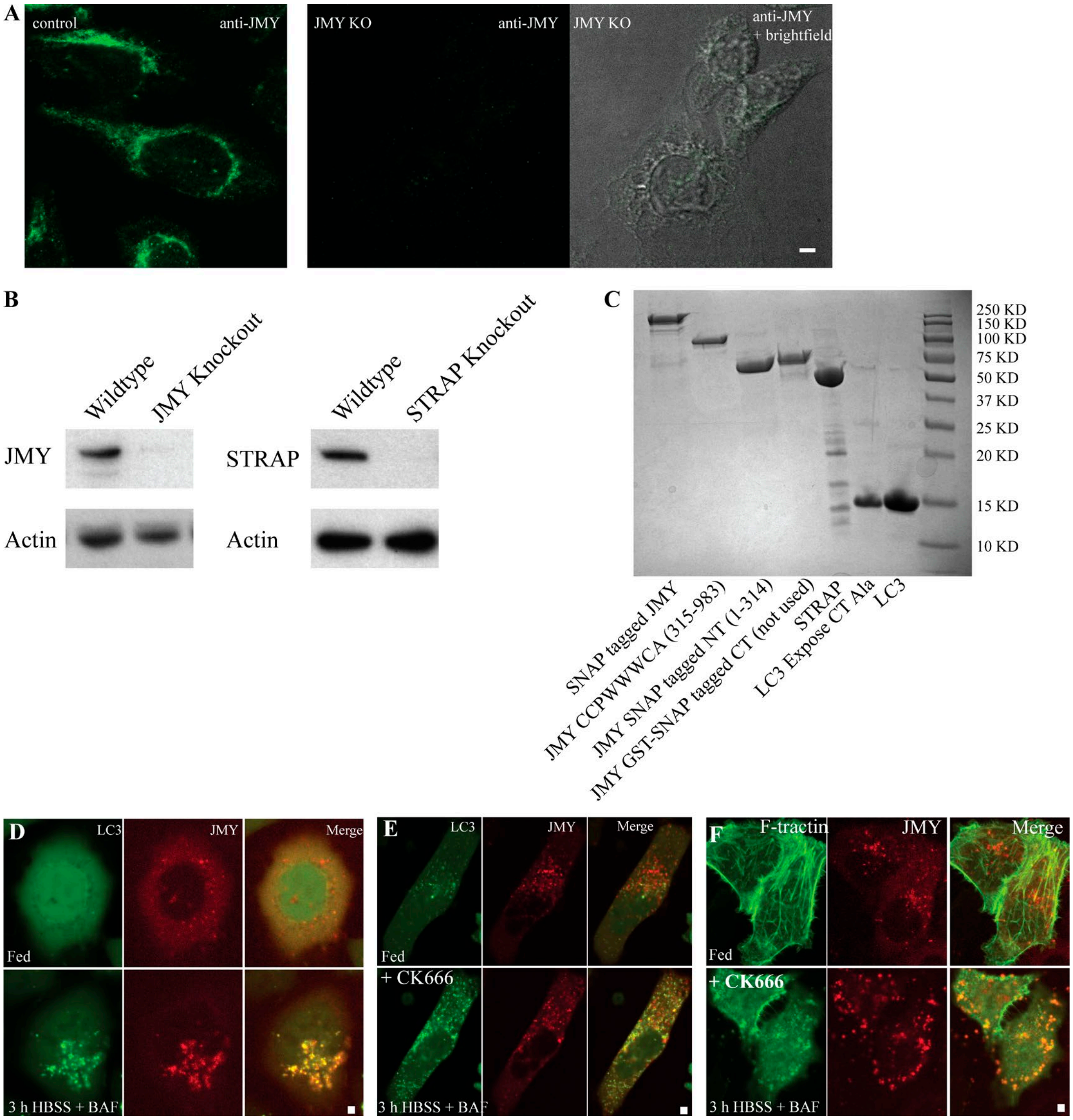

Figure S4. **CK666 inhibits the perinuclar enrichment of LC3/JMY vesicles, test of knockout cell lines, and total purified proteins used in the study.** **(A)** Immunofluorescent staining of endogenous JMY in control and JMY knockout cell line. Rabbit polyclonal serum against JMY is used as primary antibody. Scale bar: whole cell, 5 µm. **(B)** Western blot shows the knockout of JMY and STRAP in WT and JMY or STRAP knockout cell lines. **(C)** Purified proteins used in this paper on SDS-PAGE gel. **(D and E)** JMY-LC3 positive vesicles lost perinuclear enrichment when actin branched network is inhibited. (D) JMY and LC3 vesicles colocalize and comigrate to perinuclear region in cells starved and treated with 100 nM Bafilomycin A. (E) Addition of 50 µM CK666 blocks the centripetal movement of JMY/LC3 vesicles, leaving them scattered around the cytoplasm. U2OS cells were starved and treated with 100 nM Bafilomycin A. **(F)** Addition of 50 µM CK666 inhibits formation of actin branched network. Scale bars: whole cell, 5 µm.

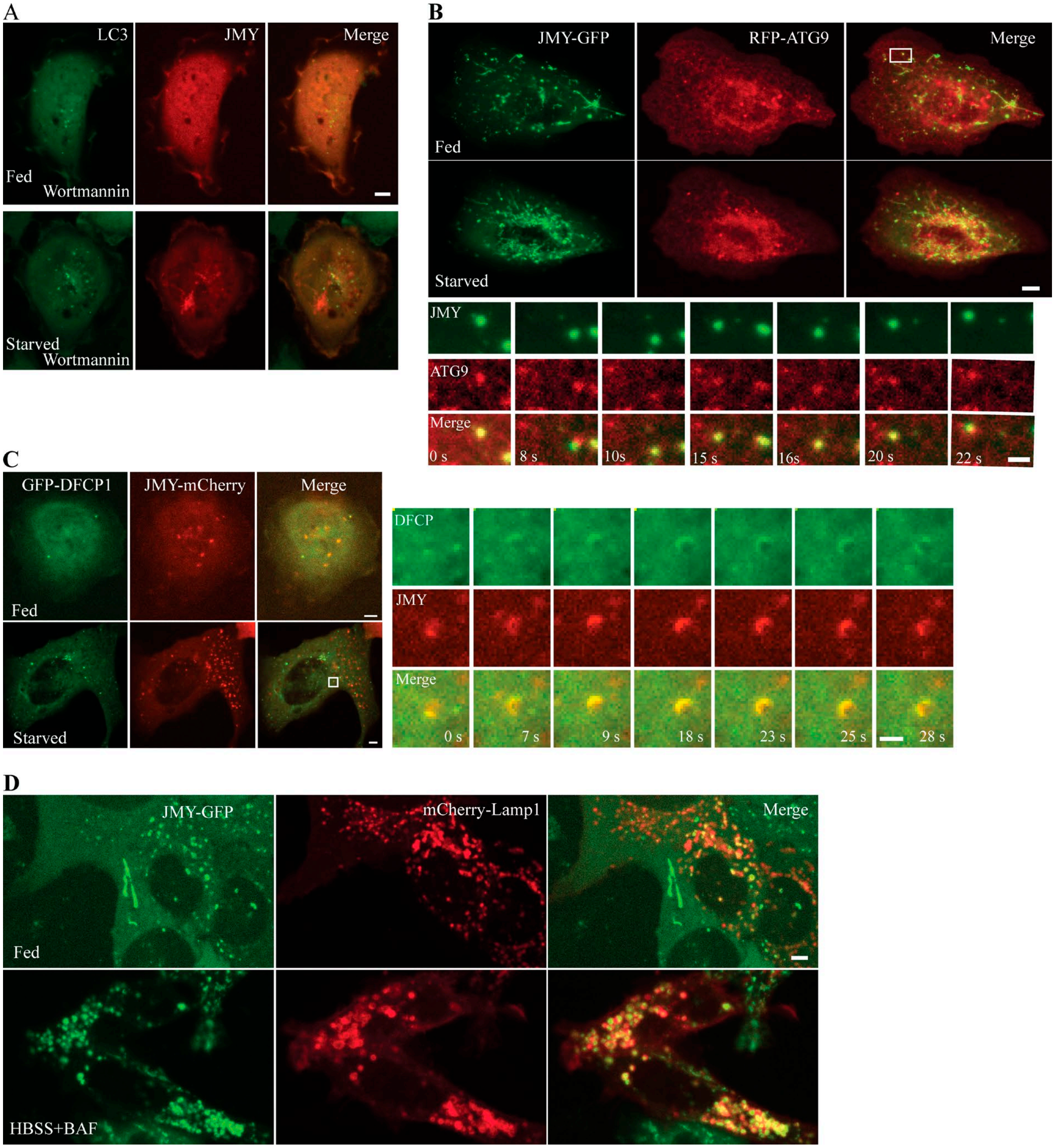

Figure S5. **Colocalization of JMY and autophagic markers. (A)** Addition of 100 nM wortmannin A inhibits formation of LC3-positive autophagosome. JMY loses its punctate structure in the cytoplasm and enriches in nucleus and plasma membrane. **(B)** JMY occasionally colocalizes and comigrates with ATG 9 puncta. **(C)** JMY occasionally colocalizes with DFCP1-labeled omegasome. **(D)** JMY colocalizes with Lamp1 when U2OS cells are starved and the autophagic flux is blocked by Bafilomycin A. Scale bars: whole cell, 5 µm; zoom-in box, 2 µm.

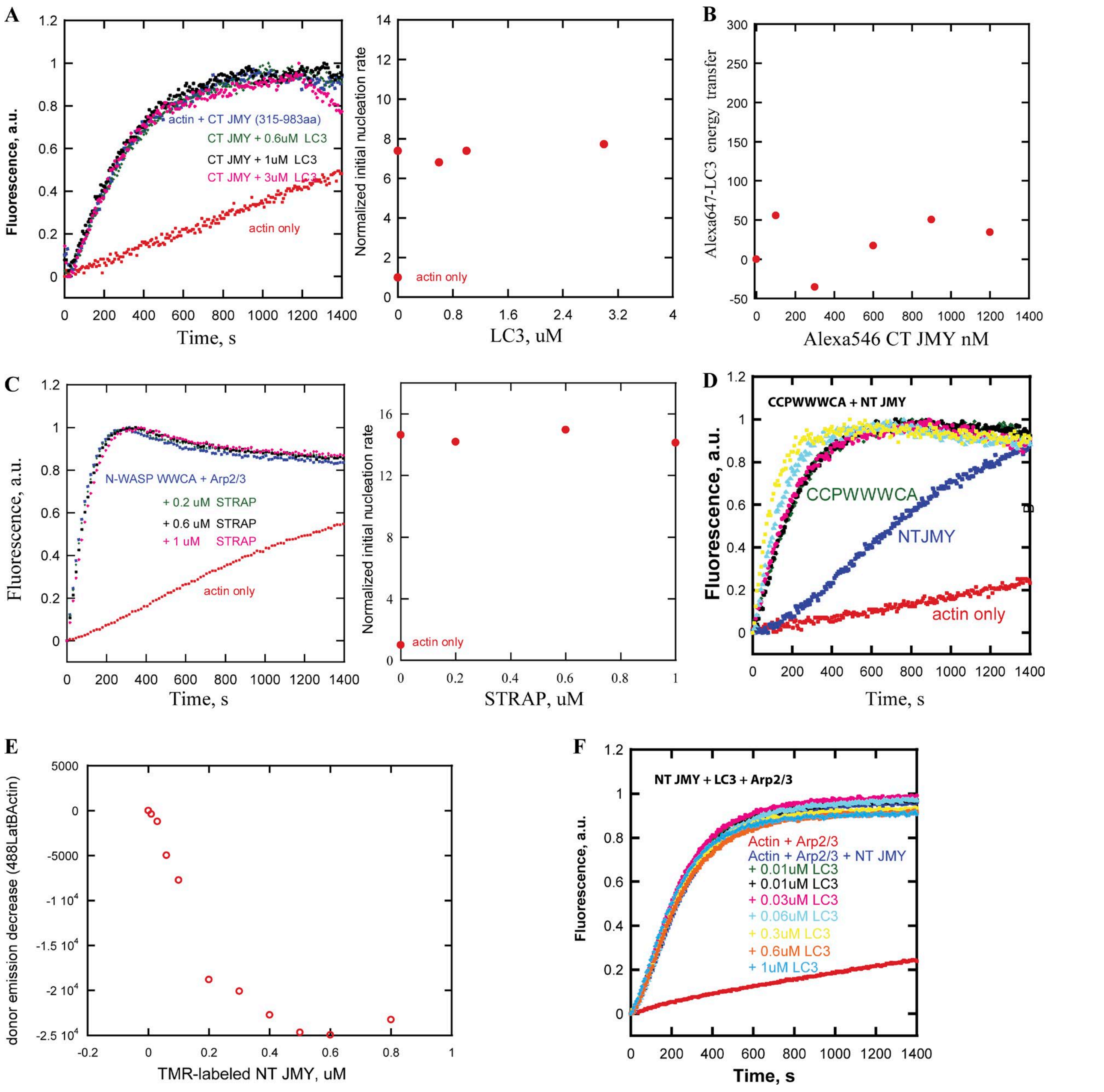

Figure S6. **Biochemical studies on regulation of JMY truncations. (A)** LC3 does not affect the nucleation activity of C terminal JMY (CCPWWWCA, residues 315–983). The initial slope (first 250 s) of each curve is normalized and plotted as a proxy for nucleation rate (middle). **(B)** LC3 does not interact with C terminal JMY (CCPWWWCA, residues 315–983) in FRET experiment. **(C)** STRAP does not inhibit Arp2/3-dependent actin nucleation activity of N-WASP WWCA domain. The initial slope (first 200 s) of each curve is normalized and plotted as a proxy for nucleation rate (middle). **(D)** Addition of N terminal JMY does not inhibit the activity of C terminal JMY, and NT JMY shows cryptic actin nucleation activity. N terminal JMY (NT JMY residues 1–314) is titrated into 200 nM C terminal JMY (CCPWWWCA, residues 315–983). Pyrene-actin polymerization assay is performed in 1× KMEI buffer with 2 µM actin. Actin only, red; actin + 500 nM NT JMY, deep blue; actin + 200 nM CCPWWWCA, green; actin + 200 nM CCPWWWCA + 100 nM NT JMY, black; +200 nM NT JMY, pink; + 500 nM NT JMY, light blue; + 1 µM NT JMY, yellow. **(E)** NT JMY (TMR-SNAP tagged, acceptor) binds latrunculin B (2 µM) actin monomer (0.1 µM Alexa Fluor 488 actin, donor) in FRET assay. **(F)** LC3 has no significant effect on NT JMY nucleation activity in the presence of Arp2/3 complex. Pyrene-actin polymerization assay is performed in 1× KMEI buffer with 2 µM actin, 200 nM NT JMY, 25 nM Arp2/3, LC3 as indicated, and 16 mM NaCl.

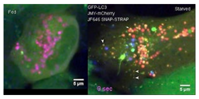

Video 1. **JMY (red) and STRAP (blue) colocalize on nonmotile puncta in fed cell (left).** Upon starvation, more JMY (red) colocalize and comigrate with LC3 (green; arrowheads; right). A subset of puncta with all three proteins (JMY, STRAP, LC3) moves in a saltatory manner (arrow). Scale bar, 5 µm.

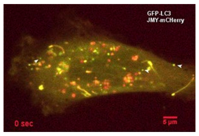

Video 2. **JMY (red) and LC3 (green) positive puncta partially colocalize and comigrate.** Scale bar, 5 µm.

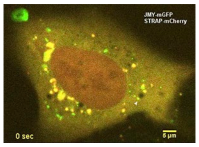

Video 3. **JMY (green) and STRAP (red) positive puncta partially colocalize and are nonmotile.** JMY- and STRAP-positive vesicles are nonmotile, and arrowheads indicate that JMY-positive and STRAP-negative vesicles are motile. Scale bar, 5 µm.

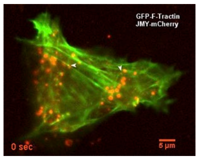

Video 4. **Polarized actin network propels JMY puncta movement.** JMY-mCherry (red) puncta travels a long distance in U2OS cell and is driven by actin network (GFP-F-Tractin; arrowhead). Scale bar, 5 µm.

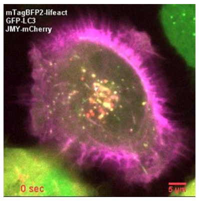

Video 5. **Polarized actin network (magenta) propels JMY (red) and LC3 (green) double positive puncta movement (arrowhead).** Scale bar, 5 µm.

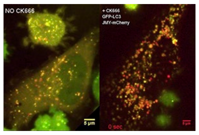

Video 6. **CK666 inhibits JMY (red) and LC3 (green) double positive puncta movement (right).** Scale bar, 5 µm.

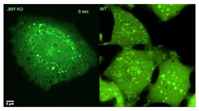

Video 7. **LC3 vesicles are much less motile in JMY knockout cell line.** GFP-LC3B vesicles are almost nonmotile in JMY knockout cells (left), whereas GFP-LC3B vesicles move in multiple directions in untreated cells (right). Scale bar, 5 µm.
